# Low‐energy electron microscopy intensity–voltage data – Factorization, sparse sampling and classification

**DOI:** 10.1111/jmi.13155

**Published:** 2022-11-30

**Authors:** Francesco Masia, Wolfgang Langbein, Simon Fischer, Jon‐Olaf Krisponeit, Jens Falta

**Affiliations:** ^1^ School of Biosciences Cardiff University Cardiff UK; ^2^ School of Physics and Astronomy Cardiff University Cardiff UK; ^3^ Institute of Solid State Physics University of Bremen Bremen Germany; ^4^ MAPEX Center for Materials and Processes University of Bremen Bremen Germany

**Keywords:** classification, hyperspectral analysis, low‐energy electron microscopy, oxide films, praseodymia, rare‐earth oxides, ruthenium dioxide, sparse sampling

## Abstract

Low‐energy electron microscopy (LEEM) taken as intensity–voltage (*I–V*) curves provides hyperspectral images of surfaces, which can be used to identify the surface type, but are difficult to analyse. Here, we demonstrate the use of an algorithm for factorizing the data into spectra and concentrations of characteristic components (FSC^3^) for identifying distinct physical surface phases. Importantly, FSC^3^ is an unsupervised and fast algorithm. As example data we use experiments on the growth of praseodymium oxide or ruthenium oxide on ruthenium single crystal substrates, both featuring a complex distribution of coexisting surface components, varying in both chemical composition and crystallographic structure. With the factorization result a sparse sampling method is demonstrated, reducing the measurement time by 1–2 orders of magnitude, relevant for dynamic surface studies. The FSC^3^ concentrations are providing the features for a support vector machine‐based supervised classification of the surface types. Here, specific surface regions which have been identified structurally, via their diffraction pattern, as well as chemically by complementary spectro‐microscopic techniques, are used as training sets. A reliable classification is demonstrated on both example LEEM *I–V* data sets.

## Lay description

Low‐energy electron microscopy (LEEM) is a powerful experimental method to image surfaces, thin films and nanoparticles. An incident beam of low energy electrons (<50eV) is reflected from the surface and used to create an image of the investigated sample. The structure of the first few atomic layers of the investigated sample is encoded in the energy dependent electron reflectivity of the surface, so called intensity versus electron energy, or in short LEEM *I* *‐V*, spectra, which however are difficult and time‐consuming to interpret.

In this paper we present a factorization method to describe the LEEM *I‐V* hyperspectral data as a combination of characteristic components which are defined by their concentrations and spectra. Using the concentration maps, we demonstrate a supervised classification method which provides a fast and reliable classification of surface reconstructions, as shown  on two examples, ruthenium oxide (RuO_2_), and praseodymium oxide (PrO_x_).

For PrO_x_, the factorization and classification reveals that the surface consists of a flat substrate with bands of coalesced oxide islands which nucleated at the atomic step edges of the Ru(0001) substrate. The PrO_x_ regions comprise a complex substructure of five distinguishable phases.

For RuO_2_, the method reveals the different types of islands that exist in the rich RuO_2_/Ru system, where different RuO_2_ orientations characteristic of the Ru oxidation can be separated by their *I‐V* spectra.

Furthermore, using the extracted component spectra and the classification of the concentrations, demonstrate a sparse sampling method to reduce the number of acquired spectral points required for classification. A reduction of the acquisition time by a factor of 30 per classification is achieved for the example data.

## 1 INTRODUCTION

Low‐energy electron microscopy (LEEM) is a powerful experimental method that provides high‐resolution hyperspectral data when used in the *I–V* imaging mode. In many applications, the information sought is the spatial distribution of surface types, that is, the structure of the first few atomic layers of the investigated sample surface. This, as well as the electronic band structure, is encoded in the energy‐dependent elastic backscattering of low‐energy electrons at the surface, so‐called intensity versus electron energy, or in short LEEM *I–V* spectra. Differing from conventional low‐energy electron diffraction intensity–voltage analysis (LEED *I–V*), where a number of diffracted beams are recorded and analysed, LEEM *I–V* analysis is usually restricted to the specular beam, that is, the (0,0) reflection in LEED, since the generation of higher‐order LEED reflections is not possible at very low electron energies due to the small radius of the Ewald sphere given by the electron wavevector. For example, the de Broglie wavelength of a 10 eV electron is 0.39 nm. This means that any scattering vector corresponding to smaller lattice dimension falls outside the Ewald sphere. At 100 eV, the electron wavelength is 0.12 nm, increasing the size of the sphere. Thus, the (0,0) reflection in LEED is the only reflection that can be expected to occur for all unfaceted coexisting surface phases regardless of their respective crystal structure.

Importantly, the resulting LEEM *I–V* spectra are difficult and time‐consuming to interpret. The dominating diffraction contrast is well understood for higher energies in the context of conventional LEED *I–V* analysis.^1^, ^2^ However, the electron energies in LEEM are in the so‐called VLEED (very low energy electron diffraction) region at energies below 50 eV. For these energies, the interaction of the electrons with the surface cannot be understood in terms of the dynamic diffraction theory which is successfully applied for higher energies.^1^, ^2^ Instead, the theoretical description is complex and electron band interactions must be taken into account.^3^, ^4^, ^5^, ^6^ Moreover, for a complete description of the data, additional contrast mechanisms like phase contrast at steps need to be considered^7^, ^8^, ^9^ and calculations for surfaces with large and inhomogeneous surface structures are not available. To circumvent this problem, a fingerprinting approach has been established already in the early 1990s.^10^, ^11^, ^12^ The term fingerprinting refers to comparing measured data with well‐known spectra (*I–V* curves). The knowledge on such spectra being a characteristic signature of a specific surface phase is usually established by employing complementary methods like X‐ray photoemission electron microscopy (XPEEM), providing chemical information, or LEED reference data of homogeneous surface regions. A typical task in a LEEM *I–V* experiment is however the study of unknown surface phases, which thus need to be identified without such prior knowledge. This is the starting point of the present study.

The task at hand is sorting the measured spectra into groups based on their similarity, at best with pixel resolution. When monitoring dynamic changes of the surface, for example by growth processes or during surface reactions, this classification should be fast and allow for real‐time data acquisition and analysis. Capturing a hyperspectral image stack with high‐energy sampling rate and sufficient signal to noise typically takes several 10 min, which is often too slow to follow the growth dynamics. Instead, a faster operation is required, which with present instrumentation can only be achieved by reducing the spectral density of the measurement, that is, reducing the number of energies measured. Furthermore, short exposure times can induce additional experimental artefacts, such as fluctuations of the electron source brightness. While such effects may be accounted for, for example by monitoring the overall intensity as a reference, it is beneficial maintaining a given exposure time and reducing the number of spectral points instead.

In the literature, classification has been done using principal component analysis of the *I–V* spectra, to reduce the dimensionality of the single pixel data, followed by unsupervised clustering algorithms like *k*‐means.^13^ This type of classification algorithm a priori assumes that each pixel is representing only one phase. Mixed phases, however, which are collected as a superposition of their components into one pixel spectrum, must be accounted for afterwards either by assignment to the most similar ‘majority’ phase, or by leaving such pixels unclassified.

**FIGURE 1:**
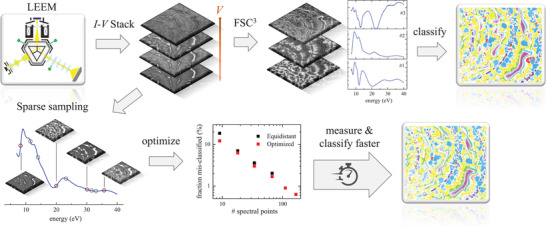
Sketch of the analysis method. LEEM *I–V* stacks are recorded, and factorized into non‐negative components and spectra by FSC^3^. Manually selected regions are used as a training set for a classifier on the resulting component concentrations. Sparse sampling retrieves the FSC^3^ concentrations from a smaller number of spectral points, down to the number of FSC^3^ components. The spectral positions are optimized for the smallest number of unclassified or misclassified points, resulting in a measurement speed‐up by an order of magnitude

We show here a method (see sketch in Figure 1) which factorizes the LEEM hyperspectral data into non‐negative components and concentrations, using the FSC^3^ algorithm.^14^, ^15^, ^16^ Using the concentrations, a supervised classification is developed using pairwise support vector machines (SVM) taking into account the uncertainty of the training set to evaluate a classification probability. This method provides a fast and reliable classification of surface reconstructions, as we show in two examples, ruthenium oxide (RuO_2_) and praseodymium oxide (PrO_*x*_).

Furthermore, using the extracted component spectra, we demonstrate a sparse sampling (SS) method similar to our previous work,^17^ but using the FSC^3^ component spectra for projection and the resulting concentrations for classification. We show that even reducing the number of spectral points to the number of components, which in our example of PrO_x_ are nine components, down from 235 in the original spectra, more than 90% of points were classified, of which less than 10% were misclassified compared to the result using all spectral points, while reducing the acquisition time by a factor of 30 per classification for the present conditions. We note that the performance is dominated by the reduction in signal‐to‐noise ratio (SNR) due to the reduced number of spectral points. The SNR is of the order of 20 for each point, limited by the shot noise of the order of 1000 electrons detected per pixel, and the typical noise factor of the micro‐channel plate amplifier of about two.^18^ We therefore expect that taking data with higher SNR, for example by a larger integration time or a higher beam current, would improve the results of the SS classifier, even when using the minimum number of spectral points.

This article is structured as follows: Section 2 provides a short explanation of the LEEM *I–V* technique, the physical interpretation of *I–V* spectra and their use in fingerprinting approaches. In Section 3, the two example data sets analysed in this study are introduced and the respective experimental details are provided. In Section 4, the data analysis procedure is presented in detail, including the pre‐processing of the data, the factorization approach, the classification technique and the optimization by SS. The results obtained on the example data sets are presented in Section 5, followed by the conclusions.

## 2 LEEM *I–V* METHODOLOGY

LEEM is a versatile technique used in surface science. It provides means for in situ structural and electronic characterisation of surfaces in ultra‐high vacuum. An electron beam with an electron energy of typically a few eV to a few tens of eV is used to homogeneously illuminate the imaged region of the sample surface. The elastically backscattered electrons pass a lens system and are projected onto the detector, forming an image. This full‐field microscopy technique enables a high time resolution of the order of 100 ms at a lateral resolution better than 10 nm. Hence, LEEM allows for observing nanoscale surface processes in real time. This can be exploited, for instance, to study surface reactions in gas atmospheres up to 10^−4^ Torr as well as growth mechanisms in molecular beam epitaxy (MBE).^19^, ^20^

A key feature of LEEM is that it can be operated in a spectro‐microscopic mode: When the electron energy is varied in small steps across a given range, a distinct intensity–voltage spectrum (*I–V* curve) can be acquired for each individual pixel of the detector camera. The *I–V* spectrum provides the energy‐dependent reflectivity of the sample surface, where contrast mainly arises from the atomic structure and the electronic band structure. At step edges and phase boundaries, phase contrast can additionally occur, and larger three‐dimensional objects like protrusions, pits and steps in the topography can cause image distortions. Depending on the diffracted order that is used for imaging, it is called bright field (using the specular (00) reflection), or dark‐field imaging (using other reflections).

During *I–V* measurements the kinetic energy of the incident electrons is varied by tuning the start voltage applied to the sample, which also affects the objective lens set‐up and hence the focussing of the obtained images. Therefore, while sweeping the start voltage the objective lens current is swept accordingly to maintain focussing.

### 2.1 Physical interpretation of *I–V* spectra

In general, solving the Schrödinger equation for the imaging electrons over the whole space of vacuum and crystal is required to generate theoretical *I–V* curves. This is needed in order to account for effects of both diffraction and electronic band structure on the electron reflection. State‐of‐the‐art ab initio calculations, using a Bloch wave ansatz in the crystal half space, are able to reproduce experimental spectra.^5^, ^6^ However, since these calculations are complex and require an accurate crystallographic model of an assumed phase a priori, the backward problem, that is, the structural and electronic characterization of unknown phases solely from their specific experimental *I–V* spectra, must be considered highly complex. As mentioned before, to bypass this, *I–V* curves can be used as a fingerprint for distinct surface phases.^21^ In this way, established fingerprints can be used to identify the phase of regions as small as the spatial resolution of the imaging.

### 2.2 Fingerprinting and classification approaches

LEEM offers several methods which allow for an assignment of *I–V* curves to structure: By adjusting the electron optics between sample and detector, it is possible to project the LEED pattern from the back focal plane of the objective lens onto the detector. Using an aperture, the surface area that constitutes the LEED pattern can be restricted down to diameters of sub‐micrometer dimensions. Thus, if large enough regions of the surface phase in question exist, information about the crystal structure can be inferred and taken into consideration for determining the phase. Furthermore, it is possible to select characteristic diffraction spots for imaging (dark‐field LEEM), which enables a highly resolved visualization of the spatial distribution of the surface components with the corresponding spatial periodicity in the crystal structure. Also, a LEEM instrument can be used in emission mode by exciting the surface with light, typically using a synchrotron as photon source to achieve high‐intensity X‐ray illumination tunable in both photon energy and incident polarization, making spectro‐microscopic methods available via photoelectron emission (XPEEM).^22^ The analysis of local X‐ray photoelectron and absorption spectra allows for a chemical analysis of the studied surface phase.

This assignment allows to establish *I–V* fingerprints for a range of surface phases. However, the identification of surface regions based on these fingerprints is often far from trivial. Firstly, the image stacks depicting a fine‐grained composition of species contain a considerable amount of edge pixels, where spectra of neighbouring phases are superimposed. Secondly, typical experiments may involve a coexistence of numerous surface phases distributed with very different area fractions. Frequently, new phases, having unknown *I–V* spectra, occur in addition to the established ones. Hence, the first challenge usually consists of dissecting the data stack into an unknown number of components, as many as there are physically distinct phases present at the surface, and elaborate their characteristic spectra therefrom.

De Jong et al.^13^ presented a method where the spectra are first reduced in dimension by principal component analysis, after which a *k*‐means clustering algorithm is employed to classify each pixel. The spectra compiled by this can then be compared to the known fingerprints to verify them. Significantly though, it is not trivial to separate an unknown number and distribution of phases in the spectral space. Because no prior information is taken into account, edge cases are not handled properly when phases superpose or when two phases have a smaller spectral variation from one another than what a third phase might encompass in itself. Also, because the *k*‐means algorithm does not employ any statistical model, evaluating the confidence in the classification requires additional analysis.

## 3 EXPERIMENTAL DATA

Here we provide details of the experimental data sets used in the analysis as exemplary data.

### 3.1 Praseodymium oxide

The PrO_*x*_ LEEM *I–V* data were recorded on an ultrathin praseodymium oxide film on a Ru(0001) surface, using the Elmitec SPELEEM at the I311 beamline^23^, ^24^ of the MAX‐lab synchrotron radiation facility in Lund, Sweden, as described in detail in Refs. 25, 26. The detector system of multi‐channel plates and camera is comparable to the one that is described in detail in Section 3.2. For the reported measurements, data were captured without binning, resulting in a 1200 pixel by 1200 pixel resolution for the full field of view. It shows a complex band‐like morphology of coalesced oxide islands, arranged along the step edges of the substrate. The Ru substrate, which is covered with an oxygen adlayer, can be identified reliably based on its characteristic *I–V* fingerprint. The praseodymia bands, on the other hand, comprise a rich substructure of coexisting surface species. Structural information was obtained from the individual phases via μ‐LEED, and X‐ray absorption spectra of the same region were collected in PEEM mode. Finally, in good agreement with theoretical reflectivity curves, five distinct praseodymium oxide phases have been characterized, with differences in stoichiometry, crystallographic structure and even surface termination. While this data set here serves to illustrate the complexity that can arise in such growth experiments, and presents a challenging test case for the presented numerical approach, the reader is referred to the original studies^25^, ^26^ for a detailed physical description of this surface system.

### 3.2 Ruthenium oxide

The RuO_2_ data set was recorded using the Elmitec SPELEEM at the Institute of Solid State Physics at the University of Bremen. The instrument is equipped with a detection assembly of multi‐channel plates and a phosphorous screen by PHOTONIS and a pco.1600 cooled charged coupled device (CCD) camera by PCO for data acquisition with low readout noise. A set of illumination apertures allows micro‐diffraction measurements on areas as small as 250 nm in diameter.

The sample was prepared in situ in the LEEM instrument. Firstly, the substrate, a commercial Ru(0001) single crystal by Mateck (<0.1° miscut), was cleaned by repeatedly oxidizing the surface with molecular oxygen and then flash‐annealing it to >1400°C.^27^ It was then subjected to atomic oxygen from an OBS 40 thermal cracker (Dr. Eberl MBE‐Komponenten GmbH). The cracker was operated at 1780°C; given the chamber geometry and the manufacturer's information, a cracking efficiency of 15% is assumed. The total atomic oxygen dose used over a duration of ca. 180 min thus amounts to 1250 L. The sample temperature was kept at 415°C during the oxidation process. After this preparation, the *I–V* LEEM image stack was acquired. For each energy in the range of 3–50 eV with a step size of 0.2 eV, a bright field image was taken with an exposure time of 4 s.

## 4 DATA ANALYSIS

LEEM *I–V* image stacks were processed using the analysis pipeline detailed in the following.

### 4.1 Image pre‐processing

At very low kinetic energies (<10 eV), the electron trajectories are strongly deflected at topographical features. In the analysis, we hence disregard images recorded in this energy regime. The energy range to be excluded depends on the respective topographical roughness of the sample region and was chosen individually for each data set, that is, <7 eV for the PrO_*x*_ data and <15 eV for the RuO_2_ data.

For the PrO_*x*_ data, containing 823 × 755 spatial pixels and 331 spectral points, we start by registering the hyperspectral data in‐plane to compensate for lateral instrumental drift, using a translation vector between two consecutive spectral frames determined by the Matlab function *imregtform*. We then denoise the data applying a singular value decomposition (SVD)^14^ on whitened data (the intensity noise in the data is dominated by electron shot noise, scaling proportional to the square root of the intensity – we thus use the square root of the data which has a noise independent of intensity, i.e. it is whitened), and retain only the 50 SVD components of highest value in the reconstructed data. We then correct for vignetting by fitting the data at 7.8 eV, which provides the most homogeneous spatial pattern (excluding outliers below 67% or above 144% of the mean) with a two‐dimensional Gaussian function. The fit is then normalized to unity centre value and the data at all energies are divided by it.

For the RuO_2_ data, containing 567 × 609 spatial pixels and 236 spectral points, we had access to measurements of the energy‐independent system sensitivity and dark current, which we used to correct the inhomogeneous illumination. The registration profile was calculated on the raw data in regions of high sensitivity. The profiles were then applied to the sensitivity corrected images. Pixels with relative sensitivity below 2% were removed. After this, SVD denoising is done as above, followed by vignetting correction using the spectrally averaged data for the fit, excluding pixels with values above 135% of the mean.

### 4.2 Factorization

After pre‐processing, the hyperspectral data are decomposed as a linear combination of components using FSC^3^, which is employing a non‐negative matrix factorization (NMF) algorithm. In this method, the hyperspectral data *D* are factorized as a linear combination of spectra *S* distributed spatially according to the maps *C*, that is D=C×S+E. The component concentrations *C* and spectra *S* are found in an iterative way, starting with random guesses, by minimizing the Fröbenius norm of the error *E*, with non‐negativity constraints on *S* and *C*. This factorization method is different from other techniques, such as PCA, where the data are expressed in terms of orthogonal components which best represent the largest variance in the data. Specifically, the orthogonality constraint results in component spectra having positive and negative values, different to physical components of intensity spectra. For the PrO_*x*_ data set, the standard FSC^3^ algorithm was used, while for the RuO_2_ data set, we used a weighted algorithm iteratively taking into account the spectral error *E*_s_ to improve retrieval of rare components, as described in Ref. 15. The spectral error is defined as the average of the square of the factorization error over the spectral domain, normalized by the average of the square of the data over both spectral and spatial domain,

(1) $$E_{s}\left(p\right)=\frac{p\sum_{s}E_{s,p}^{2}}{\sum_{s}\sum_{p}D_{s,p}^{2}},$$

where **p** indicates the number of spectral and spatial points in the image.

The weight at iteration step i+1 at spatial point *p* is taken as

(2) $$w_{i+1}\left(p\right)={\left(w_{i}\left(p\right)\frac{E_{s}\left(p\right)}{\left\langle E_{s}\right\rangle }\right)}^{1-\alpha },$$

where the ⟨.⟩ denotes the average over the spatial points, and we used α=0.5. Positions with a resulting weight exceeding $\sqrt[{4}]{p}$, were removed from the analysis in the subsequent iteration. For the PrO_*x*_ (RuO_2_) data set, nine (six) components were found to represent the data well, as judged using the spectral error showing a spatial average of around 1%. In the factorization, we have scaled the individual component concentration maps with factors minimizing the concentration error *E*_c_  defined as $|1-\sum_{k}C_{k}|$, where *k* is the component index and |.| is the 2‐norm. ^14^ The component spectra have been accordingly scaled with the inverse factors to retain the resulting factorized data.

### 4.3 Classification

The concentrations of the components obtained from the factorization are used as features for a supervised classification using an SVM. Differently from unsupervised classifiers, such as the *k‐*means used in Refs. 13, where the algorithm groups the pixels in classes depending on their similarity, the SVM method is based on training the classifier using prior knowledge. Here, the component spatial distributions are used to identify the areas of a specific class. While unsupervised classifiers do not require a training set, the number of classes needs to be determined by defining a figure of merit of the classification, with no universal accepted method available yet. For the PrO_*x*_ (RuO_2_) data, N=7 (N=5) areas were selected, each defining a class. For each pair of classes (i,j), we calculate a binary SVM classifier using the two corresponding training sets, and use it to determine, for each spatial point *p* in the image, the distance from the corresponding SVM hyperplane, $d_{\mathrm{ij}}\left(p\right)$. For each pair, we choose a distance scale to provide a mean of +1(−1) for the training sets of class i(j), respectively, and determine the resulting standard deviation $\sigma_{ij}$ of $d_{ij}$ for class *i*. Assuming a Gaussian distribution, we then calculate a probability density that a spatial point *p* is associated to class *i* as

(3) $$p_{\mathrm{ij}}\left(p\right)=\frac{1}{\sqrt{2\pi }\sigma_{i,j}}\exp\left(-\frac{{\left(d_{\mathrm{ij}}\left(p\right)-1\right)}^{2}}{2\sigma_{\mathrm{ij}}^{2}}\right).$$

The product of this density over all pairs of classes (i,j),

(4) $$P_{i}\left(p\right)=\prod_{j\neq i}p_{\mathrm{ij}}\left(p\right)$$

defines a probability density that the spatial point is associated to the class *i*. The class *l* with the largest probability density, that is $P_{l}\left(p\right)\geq P_{i}\left(p\right)$ for all classes *i*, is assigned to the spatial point *p*. To quantify the likelihood of the classification, we normalize $P_{i}\left(p\right)$ by the product of the maxima of the probability densities equation 3, yielding

(5) $${\tilde{P}}_{i}\left(p\right)=\prod_{j\neq i}\sqrt{2\pi }\sigma_{\mathrm{ij}}p_{\mathrm{ij}}\left(p\right)$$

and define the normalized average standard deviation

(6) $${\tilde{\sigma }}_{i}=\sqrt{\frac{2}{1-N}\log\left({\tilde{P}}_{i}\right)}$$

providing the average distance of the point from the training set mean in units of the training set standard deviations, quantifying the confidence of the classification. We introduce a threshold ${\tilde{\sigma }}_{t}$, to define points with ${\tilde{\sigma }}_{i}>{\tilde{\sigma }}_{t}$ as unclassified.

### 4.4 Sparse sampling

In Refs. 17, we have introduced a method to increase the acquisition speed in sequential hyperspectral imaging based on the concept of SS. In that work, we have used SVD to define a basis given by the highest singular values, and find the spectral positions of a small number of spectral points minimizing the error in the reconstruction using this basis. SVD was used as opposed to NMF to determine the basis since the reconstructed quantity was subject to further non‐linear processing prior to representing physically constrained concentrations. Here, we present a corresponding method for the LEEM data.

We use an image which covers the full spectral range sampled at the Nyquist limit of the instrument spectral resolution to determine a subset of the spectral points by minimizing a figure of demerit (FOD) related to the classification results. The FSC^3^ spectra *S* determined as detailed in Section 4.2 are used as a basis for reconstruction. A non‐denoised hyperspectral image $D^{*}$, acquired at a limited number of *N*_s_ spectral points, can be projected into the set of spectra obtained from FSC^3^ of the data with full spectral information, thus calculating the concentration distributions $C^{*}$ which can be used as features for the classifications. The concentrations are determined by solving the system $S^{*}C^{*}=D^{*}$ using a non‐negativity constraint, where $S^{*}$ is given by *S* taken at the spectral points of $D^{*}$. The classification obtained using the data with sparse sampled spectral points can differ from the classification resulting from the analysis of the data with full spectral information. To inform the choice of the spectral points, we define two FODs: (i) the fraction of points *f*_l_ with $\tilde{\sigma }>{\tilde{\sigma }}_{t}$, representing the loss of information; (ii) the fraction of misclassified points *f*_m_ relative to the classification using the full spectral information. To test the algorithm, we have divided the images into a 4 × 4 checker board pattern, where the ‘black’ fields are used to define factorization, classification and optimization of spectral points, while the ‘white’ fields serve to verify the method on an unseen data set.

We have considered different methods to determine the set of *N*_s_ spectral points: a sequential feature selection, a random sampling, a random walk sampling, a gradient‐based and a surrogate optimization algorithm.

For the sequential feature selection, we have used the MATLAB function *sequentialfs*. A forward or backward search direction can be selected. For the minimization of *f*_l_ , we have obtained similar results for both directions, while for $f_{m}$ the forward direction, that is starting with one spectral point, resulted in higher FODs than the backward direction.

In the random method, *N*_s_ spectral points are sampled with no memory of the previous step. We have considered 1000 sampling loops, and the set minimizing the FOD is returned. For the random walk approach, we maintain an approximate sampling density from the previous successful iteration. Starting with equidistant points covering the measured range, each iteration moves the points to a random position within the interval covering half the distance to the adjacent spectral points, in this way conserving the spectral ordering and the local spectral point density, while exploring the whole spectral range. Using the data $D^{*}$ at the sparse spectral points $S^{*}$, we determine the concentration maps $C^{*}$ as discussed, and classify the points using the classifier obtained from the full spectral information. The FOD's for this classification are evaluated and the new spectral points are kept for the next iteration only if the FOD was reduced. The iteration is stopped after a given number of loops – chosen to be 1000.

The gradient‐based optimization uses a ‘interior‐point’ algorithm (MATLAB function *fmincon*), where the minimum change in the spectral point for the calculation of finite‐difference gradients is set to 1. The optimization algorithm considers a continuous change in the spectral points. The minimization function includes an interpolation of both *S* and *D* for the calculation of $C^{*}$.

Finally, for the surrogate optimization we used the *surrogateopt* MATLAB function. We have set a maximum of a 1000 function evaluations.

FSC^3^ factorization and classification using the full spectral information of the black fields define our ground truth. For validation, we sample the white fields at the set of $N_{s}$ spectral points, project and classify using the same classifier, and evaluate its FOD. We have verified that using SVD denoised data for training, testing and validation data was not affecting the results significantly.

## 5 RESULTS

In this section, we present the results of the data analysis. We use the PrO_*x*_ data to demonstrate the method in detail including SS, and then provide some results for RuO_2_ to exemplify the generality of the method.

### 5.1 FSC^3^ of PrO_*x*_

**FIGURE 2:**
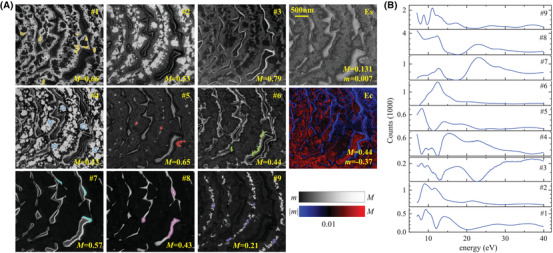
FSC^3^ analysis of LEEM *I–V* data taken on PrO_*x*_. (A) Concentrations of the components $C_{i}$, i=1…9 are given in the images on a greyscale from m=0 to *M* as labelled, together with the spectral error *E*_s_. For the latter, a logarithmic scale has been used between the given *m* and *M*. The concentration error *E*_c_ is shown on a colour scale of red (blue) hue for $E_{c}>0$ ($E_{c}<0$) as shown. The value is proportional to $\log(|E_{c}|)$, with black indicating $|E_{c}|\leq 0.01$. Each spatial region used as training set for classification is shown in one of the images, and is encircled by coloured lines. (B) Component spectra *S*

Figure 2 shows the FSC^3^ analysis of the LEEM *I–V* stack on PrO_*x*_/Ru(0001) using nine components. The sample morphology is clearly visible in these components with little noise. As characterized before,^26^ the surface consists of a flat substrate with bands of coalesced oxide islands which nucleated at the atomic step edges of the Ru(0001) substrate. In between, the bare substrate is visible in component $C_{8}$ and a transition region in the vicinity of the island edges is highlighted in $C_{3}$ and $C_{7}$. The PrO_*x*_ regions comprise a complex substructure of five distinguishable phases. In the central region, located directly at the step edges, one phase is formed as rather small, circular cores (prominent in $C_{9}$). Approaching the island edges, this central component is surrounded by a series of other distinct oxide phases: the innermost surrounding phase is visible in $C_{2}$ and $C_{4}$, a further outward one is discernible in $C_{1}$ and, finally, $C_{6}$ is located at the island rims. In addition, a rather sparse phase is identified in $C_{5}$. This component is also located primarily at the atomic step edges of the substrate as well as in a more extended, crescent‐shaped region.

In a previous analysis,^25^ the same data set was analysed by comparing the recorded spectra with curves calculated by ab initio scattering theory for a set of candidate phases. The correlation between the calculated and observed curves enabled an identification of the relevant surface phases which are visualized in the detected components. These results can be summarized as follows. The cores as well as the surrounding components, here observable in $C_{2}$, $C_{4}$ and $C_{6}$, showed only subtle differences between their respective *I–V* spectra and a reasonable agreement with the theoretical spectrum of hexagonal Pr_2_O_3_(0001). The variations of this oxide phase originate primarily from the existence of two types of terraces which are separated by atomic steps of half a unit‐cell height and feature distinct oxygen terminations. The additional separation between the cores and the outer phase prominent in $C_{1}$ was attributed to a different thickness and possibly also concomitant variations in strain relaxation and distinct surface reconstructions. Located only along the island rims, a highly oxidized fluorite PrO_2_(111) phase was identified by an *I–V* spectrum matching well to the expected theoretical spectrum. The additional sparse phase identified in $C_{5}$ was ascribed to another Pr_2_O_3_ polymorph, the cubic bixbyite‐like phase Pr_2_O_3_(111), and the spectra of the ruthenium substrate regions agree with a termination by a (1 × 1) reconstructed oxygen adlayer.

The distribution of FSC^3^ components already reflects the arrangement of these previously identified phases remarkably well – in particular considering that no laboriously precalculated spectra were necessary for this analysis. The spectral error *E*_s_ is larger in the substrate regions, whereas the concentration error *E*_c_ is large at the transition region, mostly on the left‐hand side island rims. This asymmetry might be due to an imaging error caused by not positioning the contrast aperture centred in Fourier space and does not indicate the existence of an additional physical surface component. In addition, the height change from the substrate to the islands, which was determined to be about 3–4 nm by atomic force microscopy,^26^ causes energy‐dependent deflections of the electrons. Generally, the concentration error is larger close to phase boundaries, which could be related to the coherent interference between multiple phases not accounted for by a linear mixing of intensities.

### 5.2 Classification of PrO_*x*_

**FIGURE 3:**
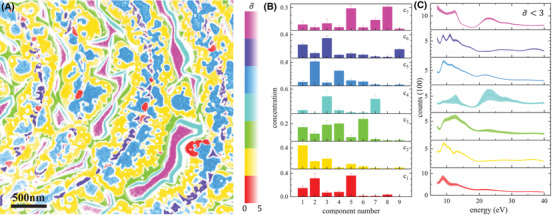
(A) Classification of LEEM *I–V* data taken on PrO_*x*_. The hue of the colour represents the assigned class *i*, with a saturation given by $\max(1-{\tilde{\sigma }}_{i}/5,0)$, indicating the assignment confidence. (B) Component concentrations with standard deviations taken from the training regions indicated in Figure 2 with the same colour coding as the classification results. (C) Class LEEM *I–V* spectra (solid lines) considering only points with $\tilde{\sigma }\leq 3$, with the shaded region indicating plus and minus the standard deviation of the spectra classified into the class region. The separate $\tilde{\sigma }$ for each class are given in the Figure S1

The FSC^3^ results shown in Figure 2 are used for a classification as described in Section 4.3. The training sets used for the classes are the areas enclosed in the colour‐coded outlines shown in selected concentration images in Figure 2. The resulting classification is given in Figure 3 with classes c_*i*_ represented by distinct hues, with a saturation encoding the classification confidence $\tilde{\sigma }$, where $\tilde{\sigma }=0$ (highest confidence) is fully saturated, dropping to zero saturation (white) for $\tilde{\sigma }\geq 5$. Again, the spatial structure of the sample is clearly visible, with the different phases discussed in Section 5.1 evident. Notably, the classes are separated by whitish regions of low classification confidence. Classes 7 and 4 are the Ru(0001)‐(1 × 1)‐O substrate and the transition regions at the island sidewalls, respectively. There is a rather sharp transition between classes 2 and 3, where the PrO_*x*_ stoichiometry was found to change,^25^ favouring the fully oxidized fluorite PrO_2_ phase at the island rims over the Pr_2_O_3_ of the central regions. Similarly, a sharp transition is observed between classes 2 and 5 on the oxide islands, reflecting the assumed change in the surface termination of the Pr_2_O_3_(0001) variations. This sharp spatial separation is remarkable as there are only rather subtle differences in the respective spectra. The island centres are consisting of classes 1 and 6, spaced by regions of low classification confidence. With respect to the FSC^3^ results in Section 5.1, an even clearer distinction between the physical surface components could be achieved. This is illustrated by the clear separation of classes 3 and 5, which were both prominent in $C_{4}$ before.

The component concentration vectors calculated as average and standard deviation over the training regions of Figure 2A are given in Figure 3A and the resulting average class spectra calculated considering only points with $\tilde{\sigma }\leq 3$ are given in Figure 3B as lines, together with their range as shaded bands. They show a large variability of c_4_, the transition region, and c_1_, the sparse Pr_2_O_3_(111) phase at the step edges, while the other classes are well defined, specifically c_2_, c_5_, c_6_ and the undisturbed substrate regions c_7_. The large variability in the transition regions between the substrate and the oxide islands can be attributed to the aforementioned imaging artefacts whose effect is dependent on the electron energy and is gradually decreasing with increasing distance to the edge of the islands, leading to the inhomogeneous transition region. On the other hand, the somewhat higher variability of c_1_, the bixbyite‐like phase, might be ascribed to an incoherent strain state within these regions, which may arise due to the underlying step edges on the substrate.

We have compared the classification results with unsupervised classifiers (see Supporting Information Section S2) including *k*‐means and Gaussian mixture models (GMM). The number of classes has been determined by Silhouette value analysis. The GMM classification obtained using features extracted by applying PCA on the SVD‐denoised data is in general agreement with our supervised approach.

The correlation‐based method using reference spectra shown in Refs. 25 provides a similar classification to Figure 3, with the major difference that only six reference spectra were considered in the original analysis, resulting in large regions of the substrate being unclassified. The FSC^3^ analysis supports the assignment of an extra training region, as seen in $C_{7}$, which allowed to classify the substrate into two classes.

### 5.3 Sparse sampling of PrO_*x*_

**FIGURE 4:**
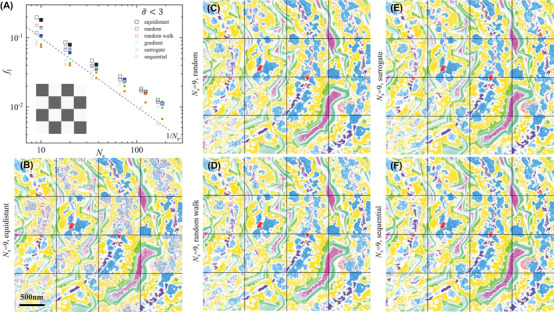
Results of the classification using sparse sampling. (A) *f*_l_ versus the number of spectral points *N*_s_ for spectrally equidistant points (black), random (blue), random walk (red), gradient‐based (green) and surrogate (magenta) optimization and sequential selection (cyan). The checker board black fields are used to define factorization, classification and optimization of spectral points, while white fields serve to verify the method on an unseen data set. The empty symbols show the FODs during the optimization procedure (black fields), while the full symbols (horizontally offset) refer to the FOD obtained when applying the method in the white validation fields. The latter are slightly displaced on the horizontal axis for visibility. The dashed line shows $1/N_{s}$. The probability threshold used was ${\tilde{\sigma }}_{t}=3$. The classification images b‐f combine the results obtained using the full spectral information (in the black fields) with the results of the sparse sampling (in the white fields) obtained either using *N*_s_= 9 equidistant points or one of the optimization methods as labelled. Colour code as Figure 3. The results for ${\tilde{\sigma }}_{t}$ of 2 and 4 using the random and random walk sampling methods are given in Figure S6

**FIGURE 5:**
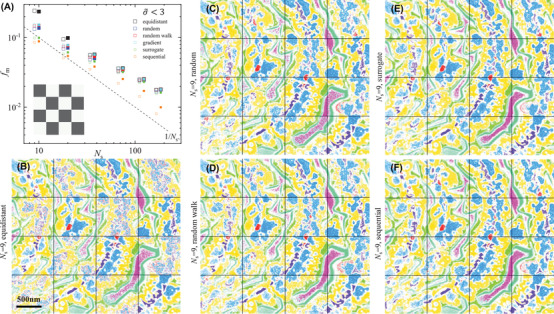
Same as Figure 4, but for the minimization of *f*_m_. The results for ${\tilde{\sigma }}_{t}$ of 2 and 4 using the random and random walk optimization methods are given in Figure S7

**FIGURE 6:**
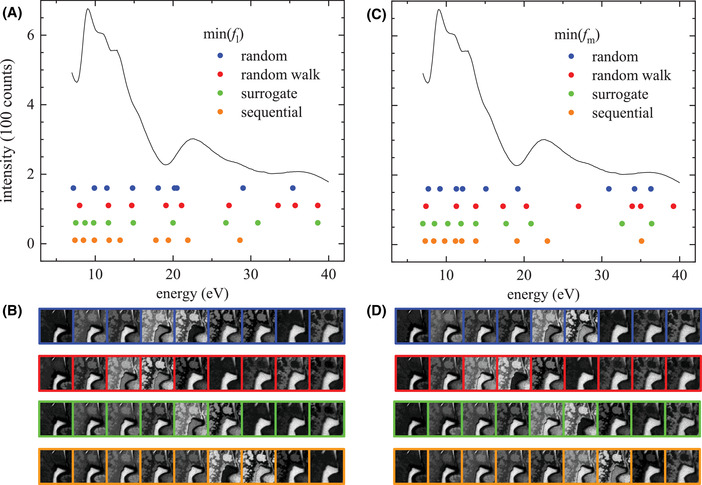
(A–C) Spectral points (symbols) obtained by minimizing the FODs, *f*_l_ (A) and *f*_m_ (C) using the random (blue), random walk (red), surrogate (green) and sequential (orange) optimization. The solid line shows the spatially averaged spectrum of the data. (B,D) The thumbnails show the LEED intensity of a selected region at the energies of the sampled spectral points, in the same horizontal order. The colour frames identify the sampling method used to minimise *f*_l_ in (B) or *f*_m_ in (D). The results use ${\tilde{\sigma }}_{t}=3$. The results for ${\tilde{\sigma }}_{t}$ of 2 and 4 for the random methods are given in Figures S16 and S17, respectively

The sparse sampling method detailed in Section 4.4 was applied to the PrO_*x*_ data, with results shown in Figures 4 and 5, using *f*_l_ and *f*_m_ as FODs, respectively. The FODs are shown in panel (a) for different number of spectral points *N*_s_. We find that generally the FODs decrease with increasing *N*_s_, as can be expected from the increasing information available. An approximate scaling of both FODs as $1/N_{s}$ is found. This indicates that the FODs are scaling with the square of the noise, as the noise is dominated by shot noise scaling as $1/\sqrt{N_{s}}$. The sequential approach provides the smallest values of the FODs among the explored optimization methods, as expected considering the deterministic nature of this feature selection. The spectral points obtained by the sequential selection are shown in Figure 6. The sampling seems to favour points in the low energy range 7–15 eV, which contains most of the variability. The evolution of the sequential selection using a backward algorithm is shown in Figure S15.

The random sampling methods reduce the values of *f*_l_ and *f*_m_ obtained by equidistant sampling by 25−40% and 40−45%, respectively. Maintaining an approximate density of spectral points, as in the random walk method, limit the possibility of sampling the low energy range appropriately, resulting in higher FODs, if compared to the fully random approach (see Figure 6). We also find that the random optimization of the spectral points is reducing the FODs stronger for smaller *N*_s_. This can be understood considering the increasing spectral separation of the points for equidistant sampling with decreasing *N*_s_, which can miss out on relevant spectral features. Adjusting the spectral positions, the points can be repositioned to sample such features better. For *N*_s_ above 100, the random optimization does not affect the FODs, while for $N_{s}=9$, the minimum number required to reconstruct the nine FSC^3^ components, the optimization improves the FODs by about a factor of 2. We also notice that optimizing for one FOD, also the other improves to some extent. The gradient‐based optimization method returns an improvement to the equidistant sampling FODs similar to the random walk approach. We speculate that this method is limited by the noise in the data which can affect the linear interpolation required in the optimization routine. The surrogate optimization performance at low *N*_s_ is instead comparable to the sequential selection, while the FODs tend to converge to the values obtained by the random and gradient‐based methods for $N_{s}≳30$, probably due to the difficulties in dealing with such large dimensionality parameter space. The evolutions of the spectral points during optimization are shown in Figures S8–S15, for the different methods and values of ${\tilde{\sigma }}_{t}$.

The resulting classifications for equidistant, as well as for *f*_m_ or *f*_l_ optimized positions, are shown for $N_{s}=9$ in Figures 4 and 5, respectively. They can be compared with the classification using all spectral points shown in Figure 3. We find that most features can be recovered by the sequential and surrogate sampling, as also suggested by the FOD values below 10%. It is important to note that the different methods come with different computational times. Table S1 gives an overview of the time needed to complete the optimizations. A good balance achieving a small FOD at low *N*_s_ and short computational expense is obtained with the surrogate optimization.

However, c_1_, which is only present in a small number of spatial points, is mostly unclassified for the equidistant spectral points, and a good classification is found only for the sequential method. The spectral points retrieved are shown in Figure 6, together with LEEM intensity images of a selected sample region at the spectral points.

The decrease in classification confidence and misclassification due to the sampling can be visualized by the ratio of the $\tilde{\sigma }$ calculated with the reduced *N*_s_ (${\tilde{\sigma }}_{\mathrm{SS}}$) and the full spectral information (${\tilde{\sigma }}_{\mathrm{Full}}$), see Figures S18–S19. As suggested by the FOD, the sequential method provides the lowest loss in confidence, as seen by the fainter colours, and less misclassified points.

This example shows that the SS allows to reconstruct spectra and classifications with a strongly reduced number of spectral points, allowing to speed up data acquisition in the present case by a factor of 33. Notably, both the fraction of non‐classified points *f*_l_, and of misclassified points *f*_m_, remain below 10% even in this case, a value limited by the SNR of the data rather than genuinely missing spectral information.

### 5.4 FSC^3^ and classification of RuO_2_

**FIGURE 7:**
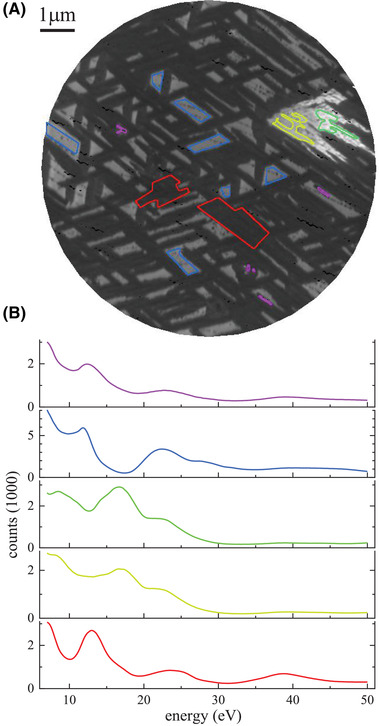
(A) LEEM image of the oxidized Ru(0001) surface taken at 19.0 eV with a field of view of 10 μm. (B) Mean *I–V* spectra extracted from the RuO_2_ data by averaging over the spatial regions as indicated by encircling lines of corresponding colour in (A), used as training set for the classification

In Figure 7A, a LEEM image from the Ru(0001) surface after oxidation is presented, showing already at a first glance the different types of islands that comprise the rich RuO_2_/Ru system. The presence of multiple different RuO_2_ orientations is characteristic for Ru oxidation and has previously been observed in PEEM and LEEM.^28^, ^29^ The formation of such oxides and their application in catalysis is reviewed in Ref. 30. To assign the islands and substrate regions observed to the corresponding structures, *I–V* fingerprint spectra as well as the μ‐LEED patterns are readily available in literature.^31^, ^32^

**FIGURE 8:**
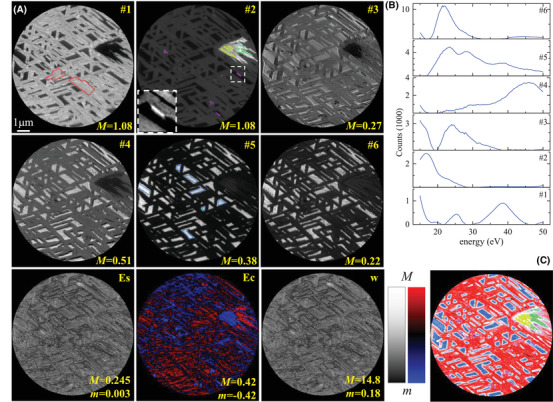
FSC^3^ analysis and classification of LEEM *I–V* data taken on a RuO_2_/Ru(0001) surface. (A) Concentrations of the components $C_{i}$, i=1…6 are given in the images on a greyscale from m=0 to *M* as labelled, together with the spectral error *E*_s_, concentration error *E*_c_ and weight *w* calculated by the weighted factorization. *E*_s_ and *w* are shown on a logarithmic scale between *m* and *M* as indicated. The concentration error *E*_c_ is shown on a colour scale red (blue) hue for $E_{c}>0$ ($E_{c}<0$), with value proportional to $\log(|E_{c}|)$, and black indicating $|E_{c}|<0.01$. The spatial regions used as training sets for classification into five classes are indicated by lines coloured according to the associated class, in the component image where they are most pronounced. The inset of $C_{2}$ shows an example of the regions used for the training of c_5_, from *m* = 0.18 to *M* = 0.28. (B) Component spectra *S*. (C) Classification results, with the hue indicating the class, and the saturation the confidence, as in Figure 3. The component concentrations over the training regions and the class spectra are shown in Figure S4, and separate $\tilde{\sigma }$ for each class are given in Figure S5

Figure 8 shows the FSC^3^ analysis (see Section 4.2) of the *I–V* LEEM stack on RuO_2_ using six components with the component spectra shown in Figure 8B. The component concentration images (Figure 8A) clearly exhibit the surface morphology expected from the single LEEM image and emphasize different parts of the surface. We notice that the weight is roughly proportional to the spectral error. This is expected considering that, at convergence, $w_{i+1}=w_{i}$, yielding (see equation 2) $w^{\alpha /(1-\alpha )}=E_{s}/\left\langle E_{s}\right\rangle$. For α=0.5, this results in a proportionality between *E*_s_ and *w*.

As in the PrO_*x*_ data, we manually select areas as indicated by the coloured lines in the component concentration images and perform a classification. The results are shown in Figure 8C and the average *I–V* spectra of each training region are presented in Figure 7B.

Most of the surface is covered by elongated structures that are about 300 nm wide, 1–5 μm long, and are aligned along three different directions. This phase has a large $C_{1}$ contribution. It exhibits the typical morphology of RuO_2_(110) islands grown below 500°C sample temperature^21^: The threefold island symmetry corresponds to the formation of three different rotational domains with the RuO_2_‐[001] crystallographic direction aligning with the primary directions of the substrate, Ru‐$\left\langle 11\bar{2}0\right\rangle$.^33^ μ‐LEED patterns taken in such sample regions (see Figure S20b) and the corresponding *I–V* spectrum (see Figure 7B) confirm this.

The classification shown in Figure 8C reliably assigns this phase in red. Only some areas at the fringe of RuO_2_(110) islands show low classification confidence – this is attributed to non‐diffraction contrast mechanisms at these islands' borders due to their height – a slight beam tilt visible in $C_{4}$ as different intensity at the edges of the islands which give a differential contrast appearance.

In $C_{6}$, some variation across the RuO_2_(110) islands' width is visible with essentially two different contrast levels. $C_{6}$ emphasizes the peak at 22 eV, which is characteristic for the substrate phase (see next paragraph). Variation in this component thus can be interpreted as a variation in the substrate signal, that is, a thickness variation of the RuO_2_(110). This indicates a decreased thickness of the RuO_2_(110) islands at their edges. As reported in Ref. 33, RuO_2_(110) growth is limited to 1.6 nm (5ML) thickness below 350°C and then gradually increases with growth temperature. In our case of 410°C growth temperature, small RuO_2_(110) islands at first only exhibit the contrast level associated with less thickness, arguably limited from further growth. After reaching a certain width, the island allows for vertical growth again and form a thicker core in their centre region.

On the right‐hand side of the data set, there is a large, arrowhead‐shaped area that has a different contrast than the above‐mentioned ones. In the growth video, this phase nucleates last and quickly grows to fill the area between the encompassing RuO_2_(110) islands. The LEED pattern (see Figure S20C) as well as the *I–V* spectrum show that this is RuO_2_(101). The island exhibits two different contrast levels as best seen in $C_{2}$, $C_{3}$ and $C_{5}$. This is because the left side of the island was subjected to a much higher electron flux at higher energies (>30 eV) during a μ‐LEED measurement series. The surrounding area was not affected as the electron beam was shielded by the illumination aperture.

The ‘substrate’, as mentioned, is most prominent in $C_{6}$ and also features in several other components, most strongly in $C_{4}$ an $C_{5}$, where the Ru step edge decorations are less pronounced. The signal weakest in $C_{1}$ and $C_{2}$, owing to those components' large emphasis on the region around 17 eV where the substrate *I–V* spectrum has a dip. In the classification image, it features as the blue phase. Based on the *I–V* spectrum in Figure 7B and the (1 × 1) reconstructed hexagonal LEED pattern (see Figure S20a), this ‘substrate’ phase can be assigned unambiguously to a one‐monolayer oxygen adlayer on Ru(0001), where each hcp hollow site is occupied by a single O atom.^6^

The adlayer‐covered substrate areas encompass small, roundish islands (diameter ≈ 10 nm) that decorate the original Ru step edges. This phase has not been captured in a separate component, due to its small surface coverage. Still, its distribution on the surface is apparent in the classification image Figure 8B inside the blue (1 × 1)‐O phase as white areas where the classification confidence is low. The close arrangement along step edges corroborates the nucleation‐and‐growth process at single steps laid out in Refs. 34 on a mesoscale. Presumably, the majority of the nuclei evolved into RuO_2_(100) islands, exhibiting the characteristic roundish shape.^35^ However, it is not possible to get conclusive LEED images from areas this small.

Interestingly, there is still another phase as revealed by the FSC^3^ analysis. Visible as highly negative values in the concentration error *E*_c_ in Figure 8A, there are small spots of somewhat irregular, elongated shape that exhibit a strongly reduced ‘concentration’. Parts of these objects do, however, show up brightly in $C_{2}$ (see inset in Figure 8A, corresponding to RuO_2_(101)) while their averaged *I–V* spectrum resembles that of RuO(110) (see Figure 7B, the red curve resembles the purple one). This indicates that they contain both orientations with grains smaller than the LEEM's lateral resolution, which in first approximation leads to a linear mixing of intensities^12^ and thus of the corresponding FSC^3^ components. The remaining concentration error then is explained by the strong faceting of some grains: Facet planes that are not parallel to the surface cannot contribute significant intensities in the specular direction, which is selected by the contrast aperture in *I–V* LEEM and represents the zeroth order of diffraction for planes normal to the optical axis. Hence, as a large surface fraction of such faceted regions remains undetected, a decrease in the overall ‘concentration’ of crystalline material in the FSC^3^ results for these islands. Furthermore, facet reflections of small inclination can yet overlap the sampled specular direction over rather broad energy intervals and hence appear as slowly varying additional background, as may be observable in the purple curve in Figure 7B at higher energies. Following these considerations, the phase is attributed to nanocrystalline RuO_2_ with diverse orientations as described by Refs. 31 and 36. It should be noted that these observations illustrate the principal difficulties which arise for any classification approach on faceted regions or high local densities of defects and adspecies. In the presented approach, the FSC^3^ concentration error provides the user with means for a facile detection of such areas where particular care must be taken in classification and physical interpretation.

## 6 CONCLUSIONS AND OUTLOOK

We have demonstrated a novel data analysis pipeline for LEEM *I–V* data. The data are represented by a few surface phase components and their spatial concentration maps. Details beyond this model are observable through analysing the factorization error in both concentration and spectrum.

For the RuO_2_ system, it could be shown that beside mesoscale RuO_2_ islands of distinct orientations, a nanocrystalline phase exists that contains RuO_2_(101) and RuO_2_(110) grains with lateral sizes below this LEEM instrument's resolution, that is, <10 nm. A very convincing classification could also be achieved for the complex example data set on PrO_*x*_  featuring a clear distinction between all pre‐characterized surface phases despite of their only subtle physical differences.

Using the concentrations, we have demonstrated a supervised classification method which classifies every point on the surface into one of the training phases, or into non‐classified if the mismatch to the training set is exceeding a given value of standard deviations.

Based on this factorization, we show a sparse sampling method using the spectral components to reconstruct full spectra from a small number of spectral points followed by classification. In the example given on the PrO_*x*_ data set, a speed‐up by a factor of 33 was achieved. This opens the perspective of real‐time classified imaging of processes on a multi‐phase surface via dynamical LEEM *I–V*. Notably, the component concentrations can also be used for unsupervised classification.

The scripts for classification and SS analysis are available at https://github.com/masiaf‐cf/leem‐svm‐classify. Information on the data underpinning the results presented here, including how to access them, can be found in the Cardiff University data catalogue at https://doi.org/10.17035/d.2022.0153725100.

## AUTHOR CONTRIBUTIONS

Wolfgang Langbein and Jens Falta conceptualized the work and methodology. Simon Fischer and Jon‐Olaf Krisponeit prepared the samples and acquired the data. Francesco Masia and Wolfgang Langbein developed the analysis software. Francesco Masia analysed the data. Simon Fischer and Jon‐Olaf Krisponeit interpreted the classification results on the exemplary data sets. Francesco Masia, Wolfgang Langbein, Jon‐Olaf Krisponeit and Simon Fischer wrote the manuscript. Jens Falta reviewed the manuscript.

## CONFLICTS OF INTEREST

The authors declare that there is no conflict of interest that could be perceived as prejudicing the impartiality of the research reported.

## Supporting information

Figure S1: Classification confidence ${\tilde{\sigma }}_{i}$ for the different classes of Pr_*x*_O, on a colour scale as given (same color scheme as Fig. 3).
Figure S2: Classification of LEEM *I‐V* data taken on PrO_x_ using *k*‐means.
Figure S3: Classification of LEEM *I‐V* data taken on PrO_*x*_ using GMM.
Figure S4: a) Component concentrations with standard deviations taken from the training regions indicated in Fig. 8 with the same colour coding as Fig. 8b).
Figure S5: Same as Fig. S1 for the RuO_2_ dataset.
Figure S6: Additional results of the classification using sampled energies with a probability threshold ${\tilde{\sigma }}_{t}$ = 2 (a‐c) and ${\tilde{\sigma }}_{t}$ = 4 (d‐f) and the random (b,e) and random walk (c,f) optimisation to minimise $f_{l}$.
Figure S7: Same as Fig. S6 for the minimisation of $f_{m}$.
Figure S8: a‐b) Evolution of the spectral points positions (a) and $f_{l}$ (b) during fully random sampling for $N_{S}$ = 9 and minimisation of $f_{l}$ for a probability threshold of ${\tilde{\sigma }}_{t}$ = 3.
Figure S10: Same as S8 for a probability threshold of ${\tilde{\sigma }}_{t}$ = 4.
Figure S11: Same as S8 for the random walk optimisation and a probability threshold of ${\tilde{\sigma }}_{t}$ = 3.
Figure S12: Same as S8 for the random walk optimisation and a probability threshold of ${\tilde{\sigma }}_{t}$ = 2.
Figure S13: Same as S8 for the random walk optimisation and a probability threshold of ${\tilde{\sigma }}_{t}$ = 4.
Figure S14: Same as S8 for the surrogate optimisation and a probability threshold of ${\tilde{\sigma }}_{t}$ = 3.
Figure S15: a‐b) Evolution of the sequential selection of the spectral points positions (a) and *f*_1_ (b) for $N_{S}$ = 9 and minimisation of *f*_1_ for a probability threshold of ${\tilde{\sigma }}_{t}$ = 3.
Figure S16: Spectral positions of the points optimised by the random (a,c) and random walk (b,d) methods for probability threshold of ${\tilde{\sigma }}_{t}$ = 2.
Figure S17: Same as Fig. S16 for ${\tilde{\sigma }}_{t}$ = 4.
Figure S18: Loss of classification between sparse sampling and full spectral information.
Figure S19: Same as Fig. S18 for the minimisation of $f_{m}$.
Figure S20: Low‐energy electron diffraction (μ‐LEED) patterns taken from specific regions on the RuO_2_ sample presented in the main manuscript.
