# Supplementary material for: Low‐energy electron microscopy intensity–voltage data – Factorization, sparse sampling and classification
Source: J Microsc. 2022 Nov 30;289(2):91–106. doi: 10.1111/jmi.13155 (PMC10108219; doi:10.1111/jmi.13155)
Supplement: Supplementary file 1 — Figure S1: Classification confidence σ∼i for the different classes of Pr x O, on a colour scale as given (same color scheme as Fig. 3). Figure S2: Classification of LEEM I‐V data taken on PrOx using k‐means. Figure S3: Classification of LEEM I‐V data taken on PrO x using GMM. Figure S4: a) Component concentrations with standard deviations taken from the training regions indicated in Fig. 8 with the same colour coding as Fig. 8b). Figure S5: Same as Fig. S1 for the RuO2 dataset. Figure S6: Additional results of the classification using sampled energies with a probability threshold σ∼t = 2 (a‐c) and σ∼t = 4 (d‐f) and the random (b,e) and random walk (c,f) optimisation to minimise fl. Figure S7: Same as Fig. S6 for the minimisation of fm. Figure S8: a‐b) Evolution of the spectral points positions (a) and fl (b) during fully random sampling for NS = 9 and minimisation of fl for a probability threshold of σ∼t = 3. Figure S10: Same as S8 for a probability threshold of σ∼t = 4. Figure S11: Same as S8 for the random walk optimisation and a probability threshold of σ∼t = 3. Figure S12: Same as S8 for the random walk optimisation and a probability threshold of σ∼t = 2. Figure S13: Same as S8 for the random walk optimisation and a probability threshold of σ∼t = 4. Figure S14: Same as S8 for the surrogate optimisation and a probability threshold of σ∼t = 3. Figure S15: a‐b) Evolution of the sequential selection of the spectral points positions (a) and f 1 (b) for NS = 9 and minimisation of f 1 for a probability threshold of σ∼t = 3. Figure S16: Spectral positions of the points optimised by the random (a,c) and random walk (b,d) methods for probability threshold of σ∼t = 2. Figure S17: Same as Fig. S16 for σ∼t = 4. Figure S18: Loss of classification between sparse sampling and full spectral information. Figure S19: Same as Fig. S18 for the minimisation of fm. Figure S20: Low‐energy electron diffraction (μ‐LEED) patterns taken from specific regions on the RuO2 sample presented in [file JMI-289-91-s001.pdf]

Low-energy electron microscopy intensity-voltage data – factorization, sparse sampling, and classification – Supplementary Information

F. Masia[1,2], W. Langbein[2], S. Fischer[3], J.-O. Krisponeit[3,4], and J. Falta[3,4]

[1]School of Biosciences, Cardiff University, Museum Avenue, Cardiff CF10 3AX, United Kingdom
[2]School of Physics and Astronomy, Cardiff University, The Parade, Cardiff CF24 3AA, United Kingdom
[3]Institute of Solid State Physics, University of Bremen, Otto-Hahn-Allee 1, 28359 Bremen, Germany
[4]MAPEX Center for Materials and Processes, University of Bremen, 28359 Bremen, Germany

S1 Classification probabilities for PrO_x

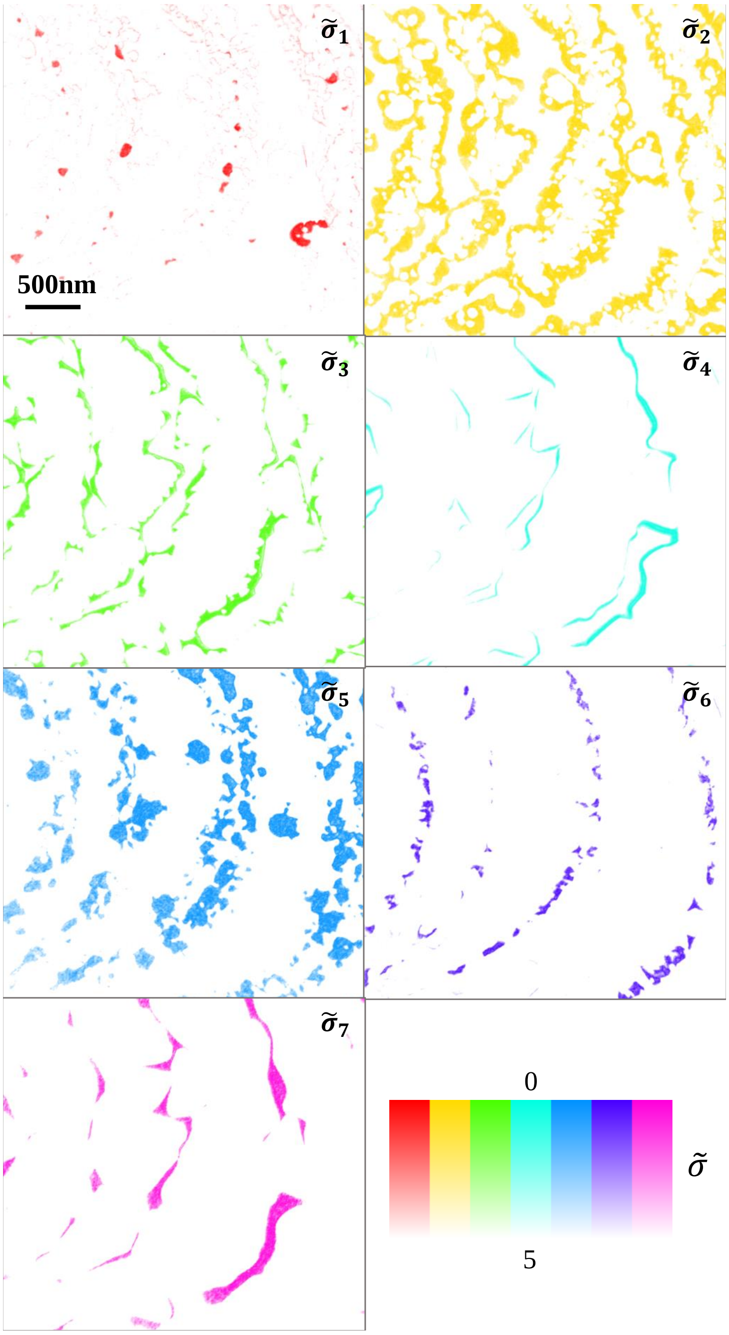

Figure S1: Classification confidence $\tilde{\sigma}_i$ for the different classes of PrO_x, on a colour scale as given (same color scheme as Fig. 3).

S2 Unsupervised classification of PrO_x data

We have compared our supervised method with other unsupervised approaches (*k-means* and GMM, Gaussian Mixture Models) to classify the PrO_x data. The features used in the classification are defined by either the FSC^3 or a standard PCA of the hyperspectral data. In the latter case, either raw or SVD-denoised data have been considered, and only the highest weight components retaining 99% of the variance have been included, corresponding to 10 and 6 components for the raw and denoised data, respectively. To determine the number of classes we have calculated the Silhouette value for increasing number of classes (from 2 to 10) and considered the classification maximising the average Silhouette value, as a way to identify compact clustering. Fig. S2 shows the results of a *k-means* classifier applied to the features extracted by FSC^3 (panel a) and PCA (raw data, panel c, and de-noised data, panel e). Five replica with different initial cluster centroids have been considered, and we used the solution with the lowest within-cluster sums of point-to-centroid distances. The number of classes maximising the Silhouette values were 6, 6 and 8, respectively. In all three cases, the classification is dominated by fluctuations in the substrate which is divided in several classes, while not all the PrO_x phases are retrieved. Similarly, Fig. S3 shows the results of the GMM classifications for the different input features. Here, we have used full covariance matrices for all the classes. Five replica with different initial positions of the Gaussian distributions have been considered. For the calculation of the Silhouette values, the pixels have been assigned to the class with the highest posterior probability. The number of classes obtained for the different input features were 5, 6 and 7, respectively. Fig. S3 shows the values of $\tilde{\sigma}$ as defined in Sec. 4.3, using the posterior probability for $\tilde{P}$. The GMM method does not seem to be affected strongly by the fluctuations in the substrate and most of the phases of the PrO_x observed in the FSC^3 are retrieved. The GMM classification of features obtained from PCA on the de-noised data is similar to the result of our supervised approach. The calculation of the Silhouette values for the different number of classes is slow. The total computational time (using two 6-core Intel Xeon E5-2620 V3 CPU) for the GMM classifications using PCA on denoised data and number of classes from 2 to 10 was $\sim$ 156 minutes, $\sim$ 4 orders of magnitude slower than the supervised classifier.

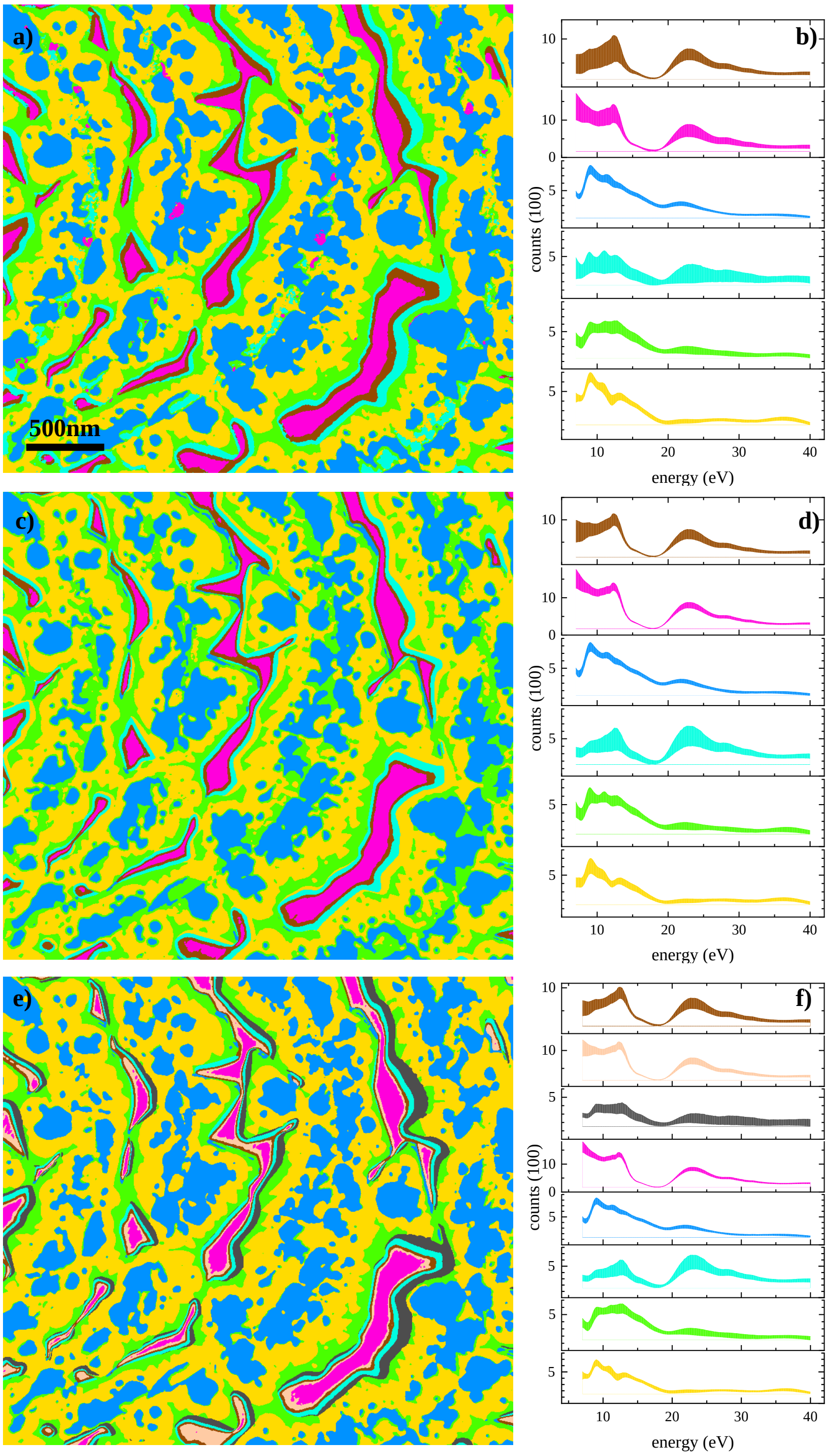

Figure S2: Classification of LEEM I-V data taken on PrO_x using *k-means*. The hue of the colour represents the assigned class. The features used in the classification are obtained by a) FSC[3] c) PCA on noisy data and e) PCA on SVD-denoised data. The spectra observed over the class region are shown in panels b), d) and f), for the different classifications, with the shaded region representing the averaged spectra ± the standard deviation.

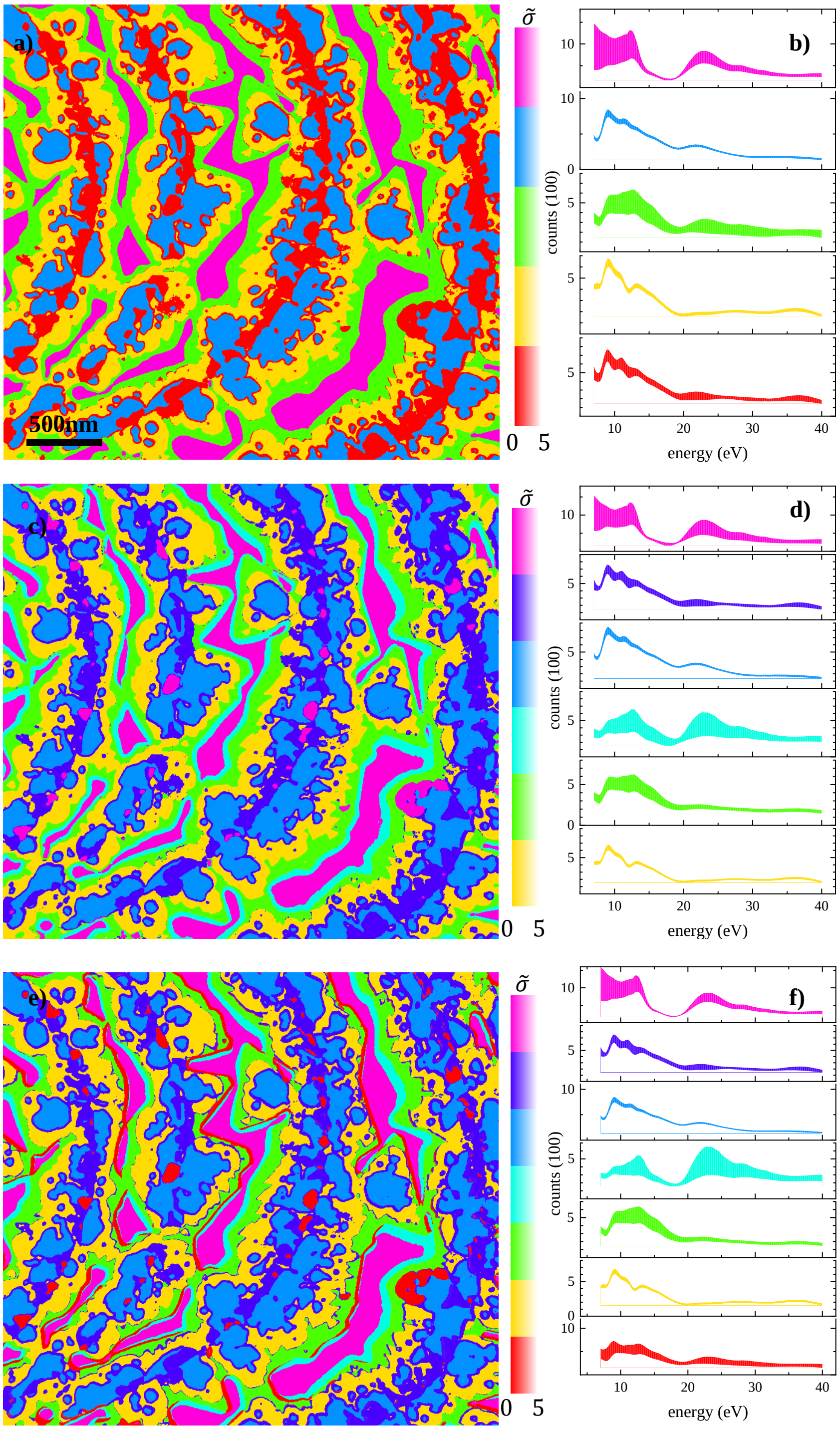

Figure S3: Classification of LEEM I-V data taken on PrO_x using GMM. The hue of the colour represents the assigned class, with a saturation given by $\max(1-\tilde{\sigma}_i/5, 0)$. The features used in the classification are obtained by a) FSC[3] c) PCA on noisy data and e) PCA on SVD-denoised data. The spectra observed over the class regions considering only the pixels with $\tilde{\sigma} \leq 3$ are shown in panels b), d) and f), for the different classifications, with the shaded region representing the averaged spectra ± the standard deviation.

S3 Class component concentrations and spectra for RuO_2

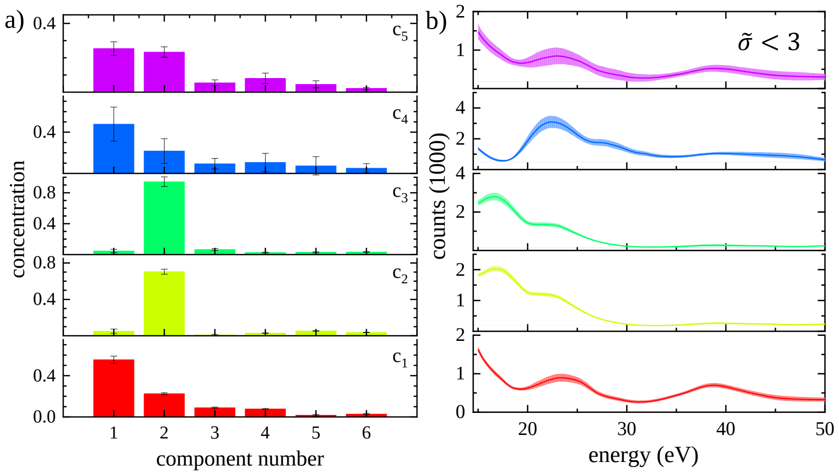

Figure S4: a) Component concentrations with standard deviations taken from the training regions indicated in Fig. 8 with the same colour coding as Fig. 8b). b) Class spectra (solid lines) considering only the pixels with $\tilde{\sigma} \leq 3$, with the range of spectra classified into the class given as shaded region.

S4 Classification probabilities for RuO_2

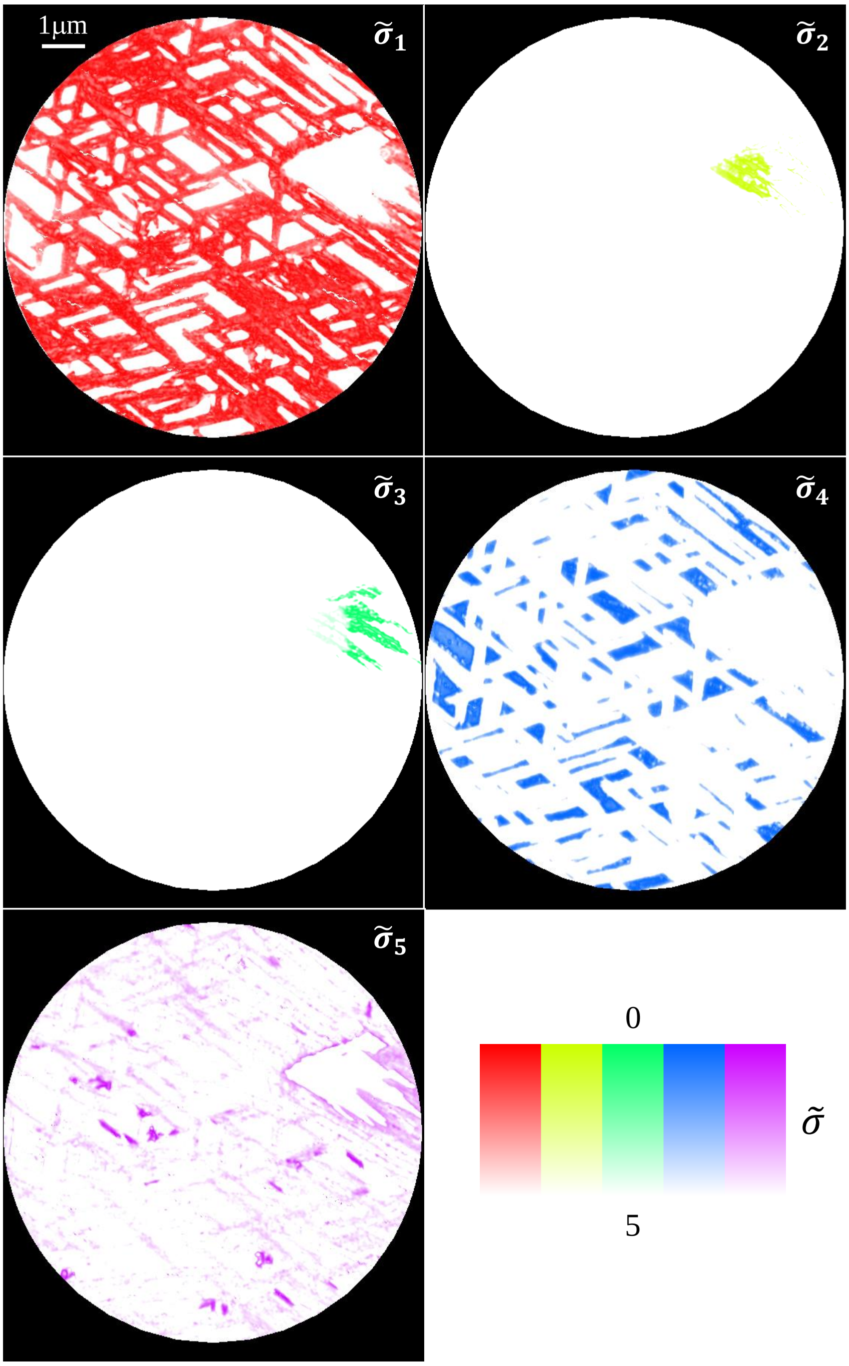

Figure S5: Same as Fig. S1 for the RuO_2 dataset.

Table S1: Computational times for the different sampling methods as discussed in the main manuscript for $N_s = 9$, using a AMD Ryzen 9 5900X 12 core CPU. [p] indicates parallel computation.

Method	Time (s)
Equidistant	4.2
Random (1000 iterations) [p]	1018
Random walk (1000 iterations)	2098
Gradient	525
Surrogate (1000 iterations) [p]	6526
Sequential (forward) [p]	30782

S5 Sparse Sampling

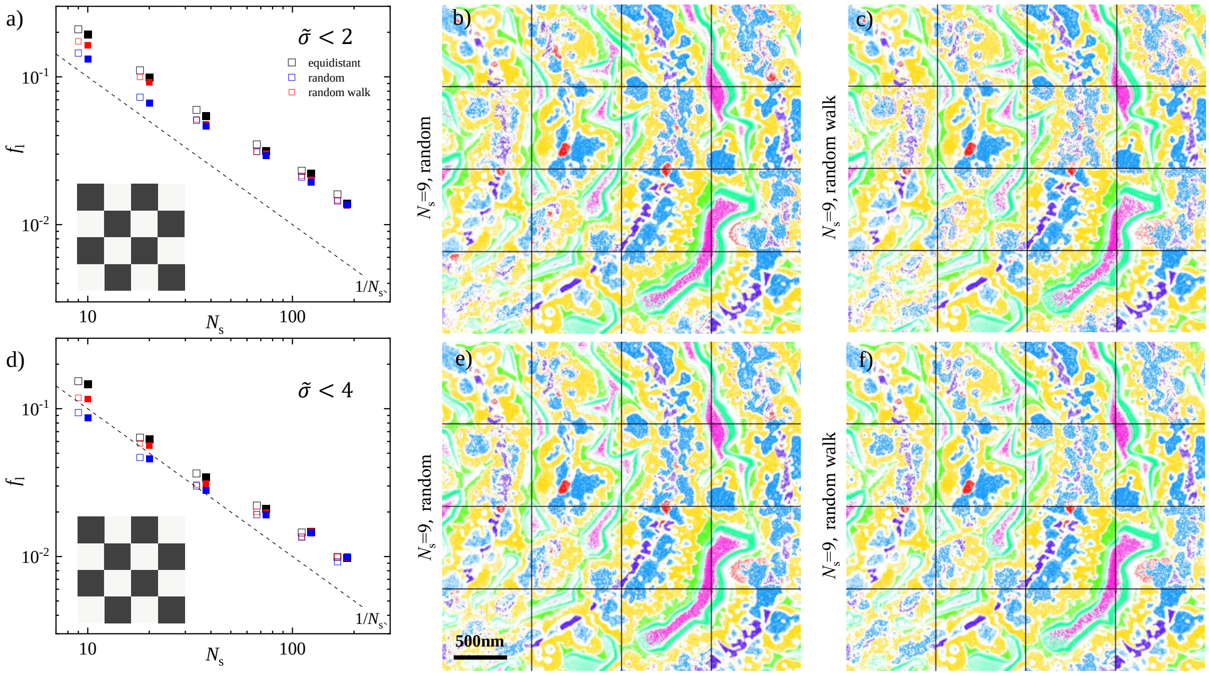

Figure S6: Additional results of the classification using sampled energies with a probability threshold of $\tilde{\sigma}_t = 2$ (a-c) and $\tilde{\sigma}_t = 4$ (d-f) and the random (b,e) and random walk (c,f) optimisation to minimise f_l. The classification maps use the same hue and saturation encoding as Fig. 3

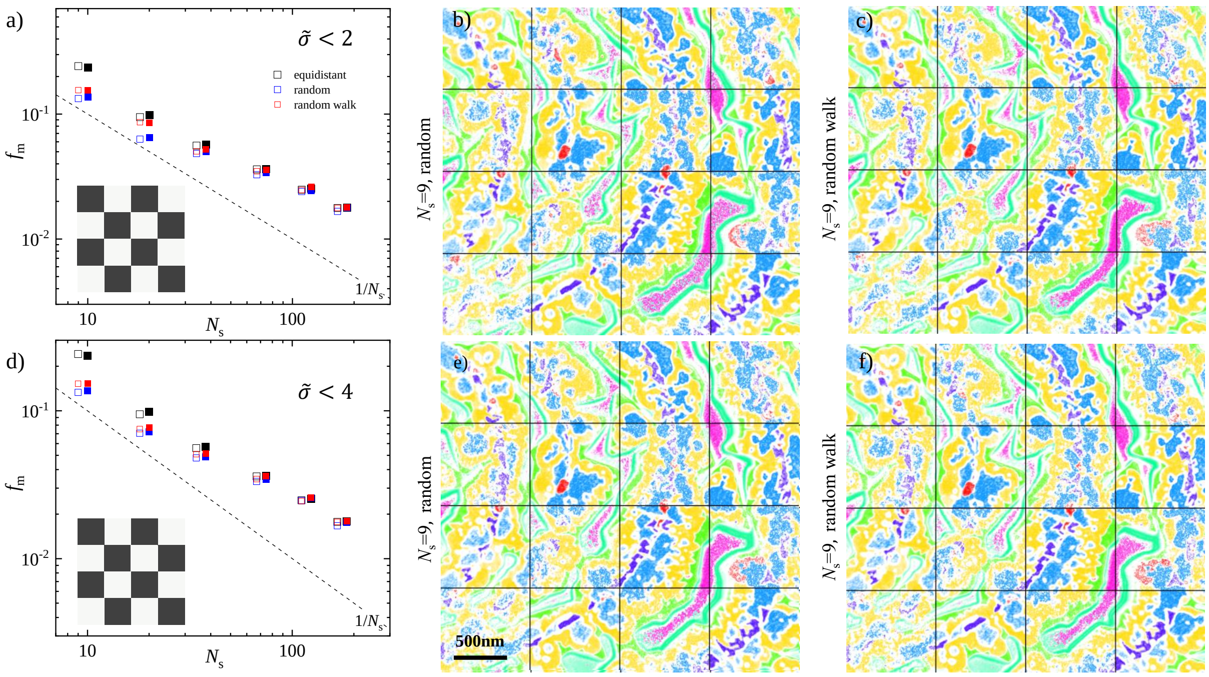

Figure S7: Same as Fig. S6 for the minimisation of f_{m}.

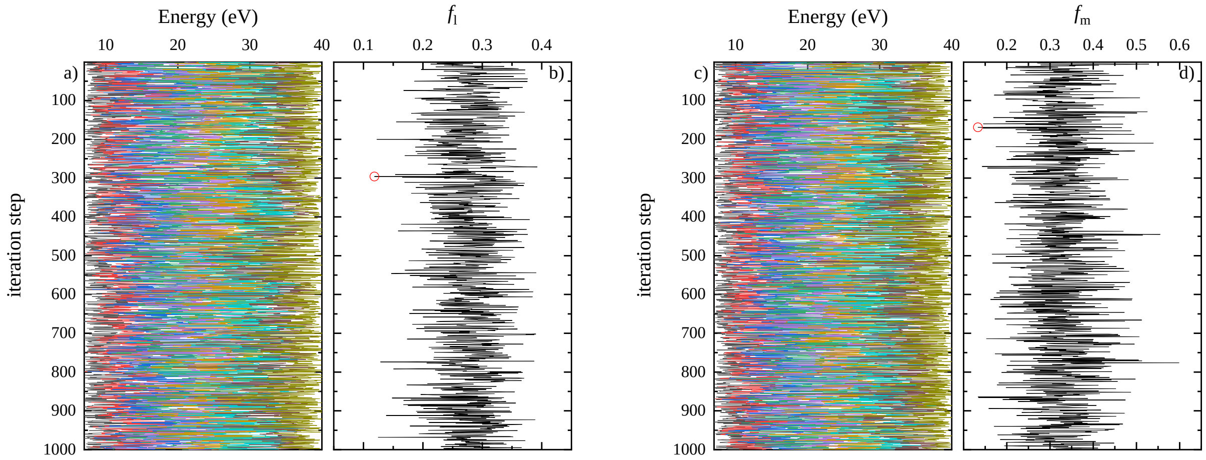

Figure S8: a-b) Evolution of the spectral points positions (a) and f_{l} (b) during fully random sampling for $N_{\mathrm{s}} = 9$ and minimisation of f_{l} for a probability threshold of $\tilde{\sigma}_{\mathrm{t}} = 3$. The red circle indicates the minimum FOD obtained. c-d) Same as a-b) for the minimisation of f_{m}.

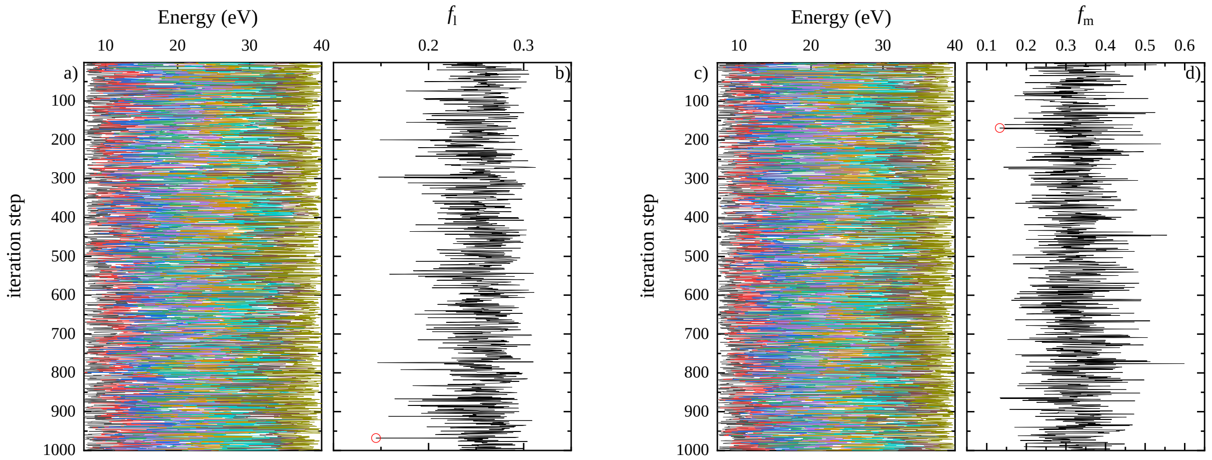

Figure S9: Same as S8 for a probability threshold of $\tilde{\sigma}_t = 2$.

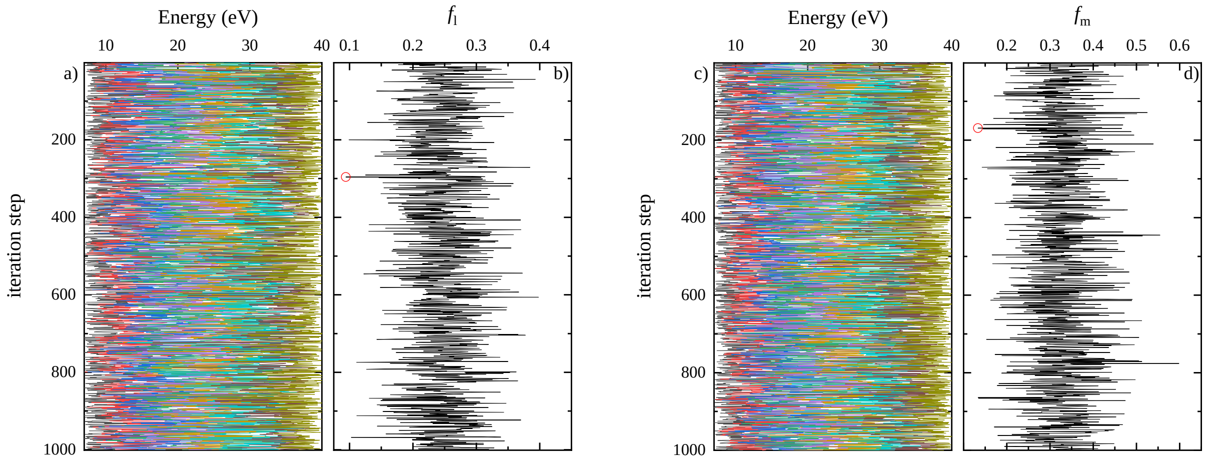

Figure S10: Same as S8 for a probability threshold of $\tilde{\sigma}_t = 4$.

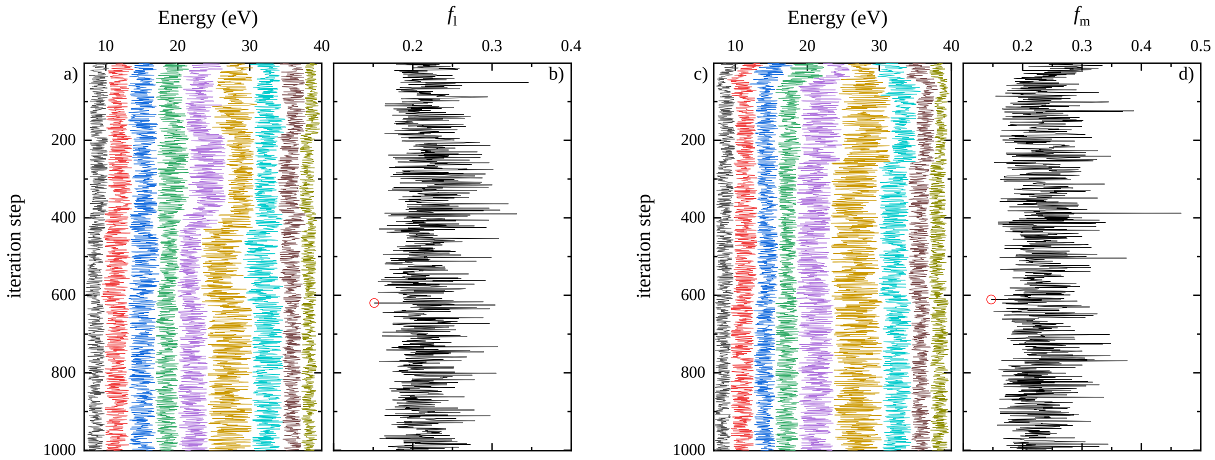

Figure S11: Same as S8 for the random walk optimisation and a probability threshold of $\tilde{\sigma}_t = 3$.

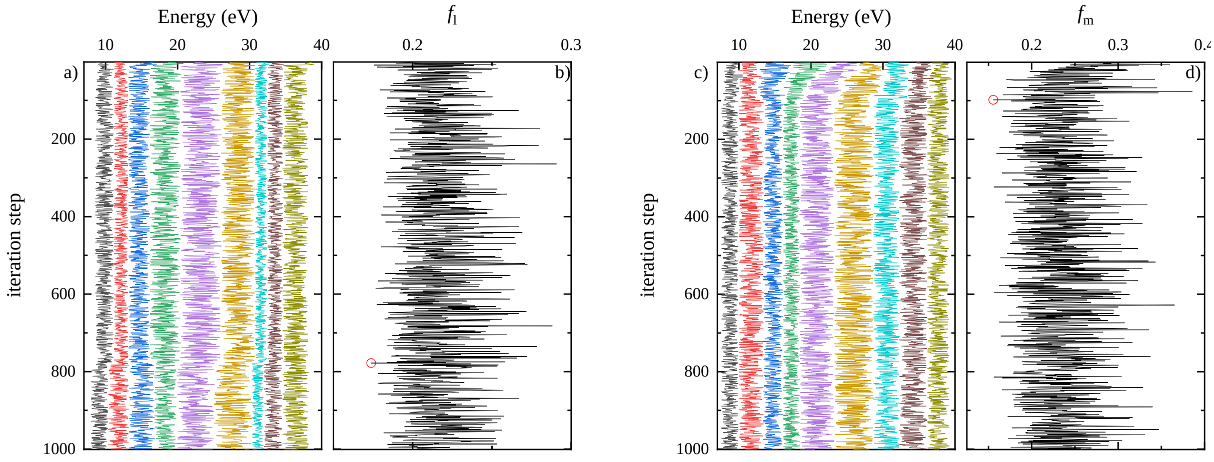

Figure S12: Same as S8 for the random walk optimisation and a probability threshold of $\tilde{\sigma}_t = 2$.

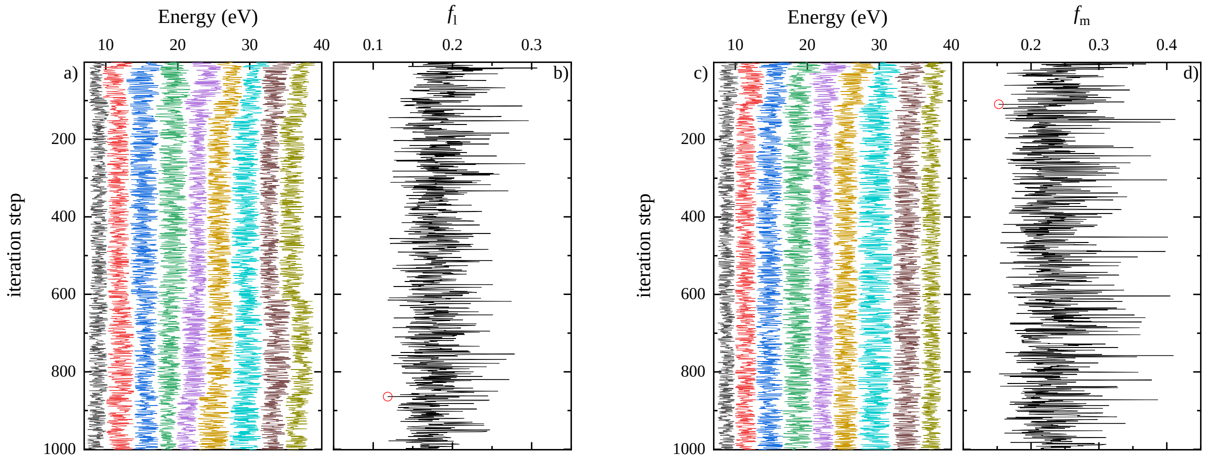

Figure S13: Same as S8 for the random walk optimisation and a probability threshold of $\tilde{\sigma}_t = 4$.

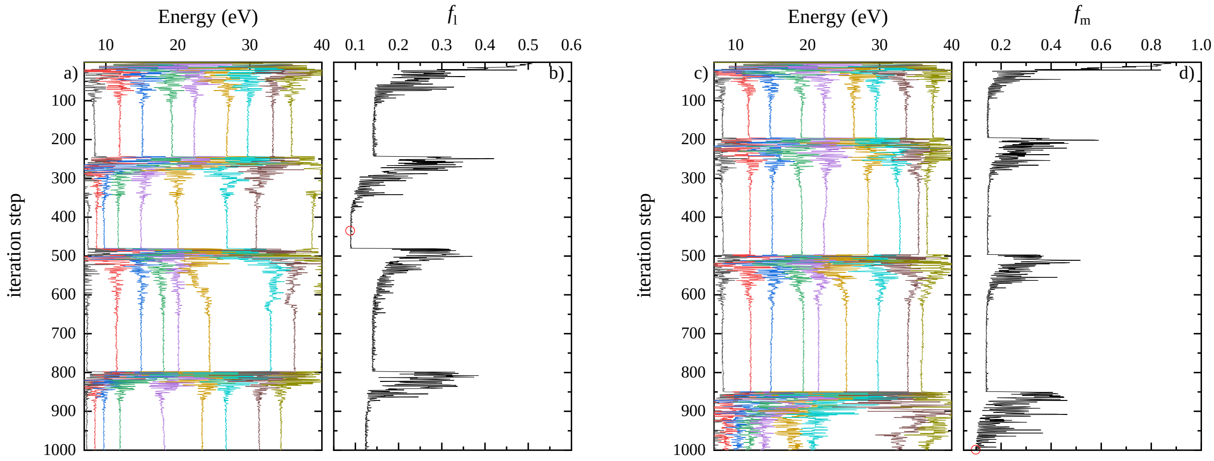

Figure S14: Same as S8 for the surrogate optimisation and a probability threshold of $\tilde{\sigma}_t = 3$.

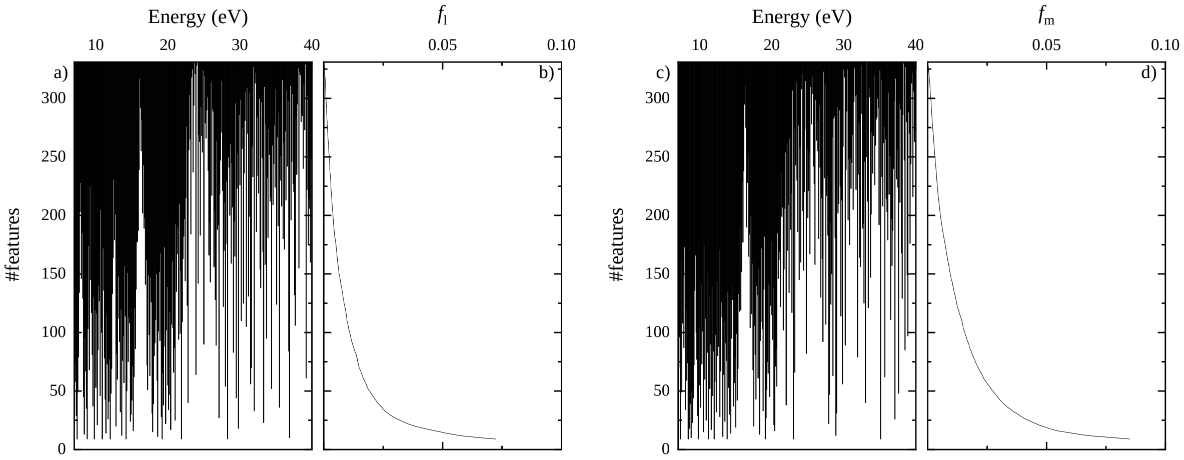

Figure S15: a-b) Evolution of the sequential selection of the spectral points positions (a) and f_l (b) for $N_s = 9$ and minimisation of f_l for a probability threshold of $\tilde{\sigma}_t = 3$. c-d) Same as a-b) for the minimisation of f_m.

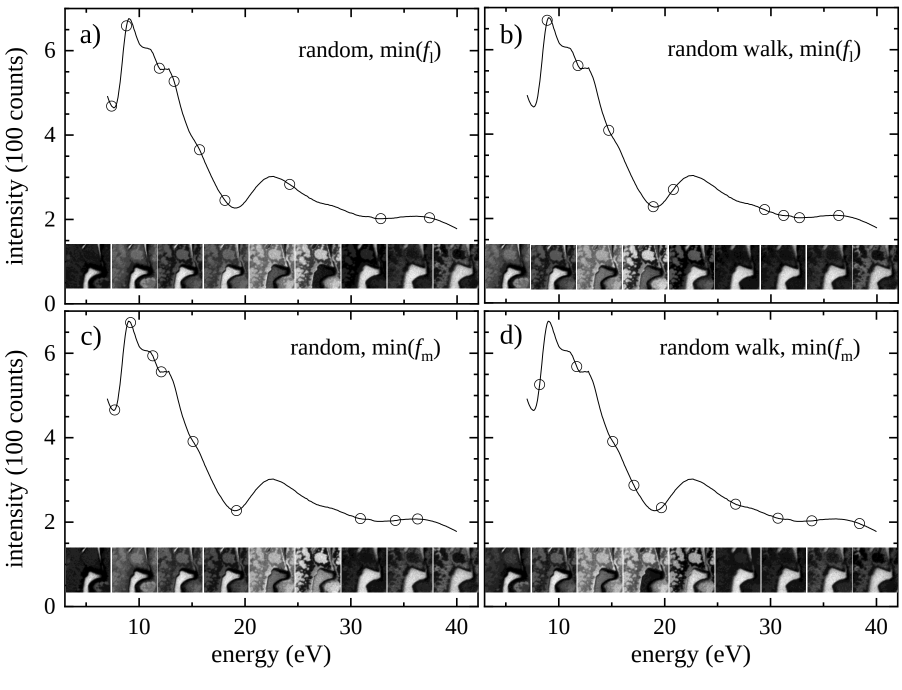

Figure S16: Spectral positions of the points optimised by the random (a,c) and random walk (b,d) methods for probability threshold of $\tilde{\sigma}_t = 2$. (a,b) and (c,d) shows the result of the minimisation of f_l and f_m, respectively.

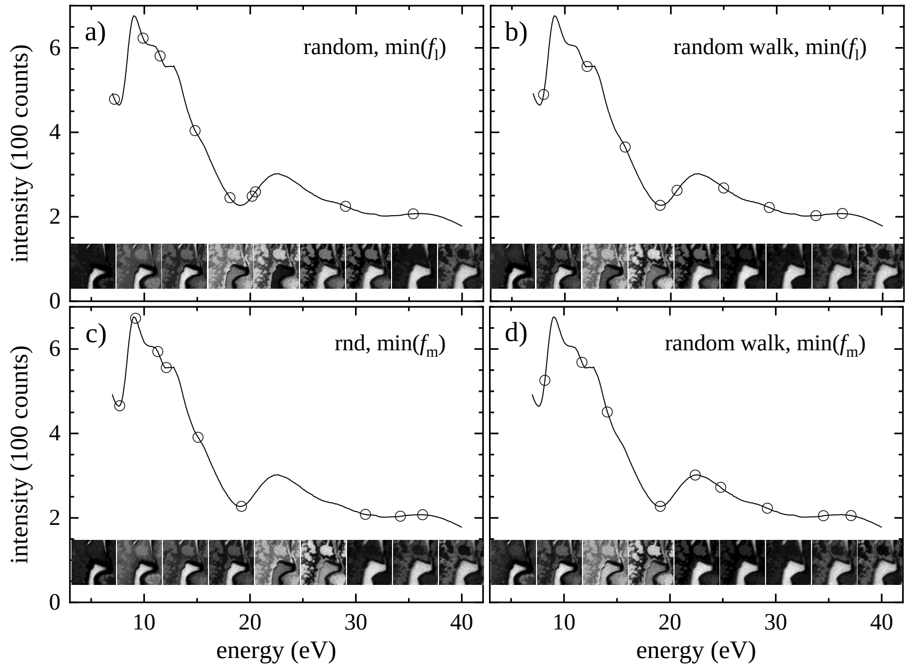

Figure S17: Same as Fig. S16 for $\tilde{\sigma}_t = 4$.

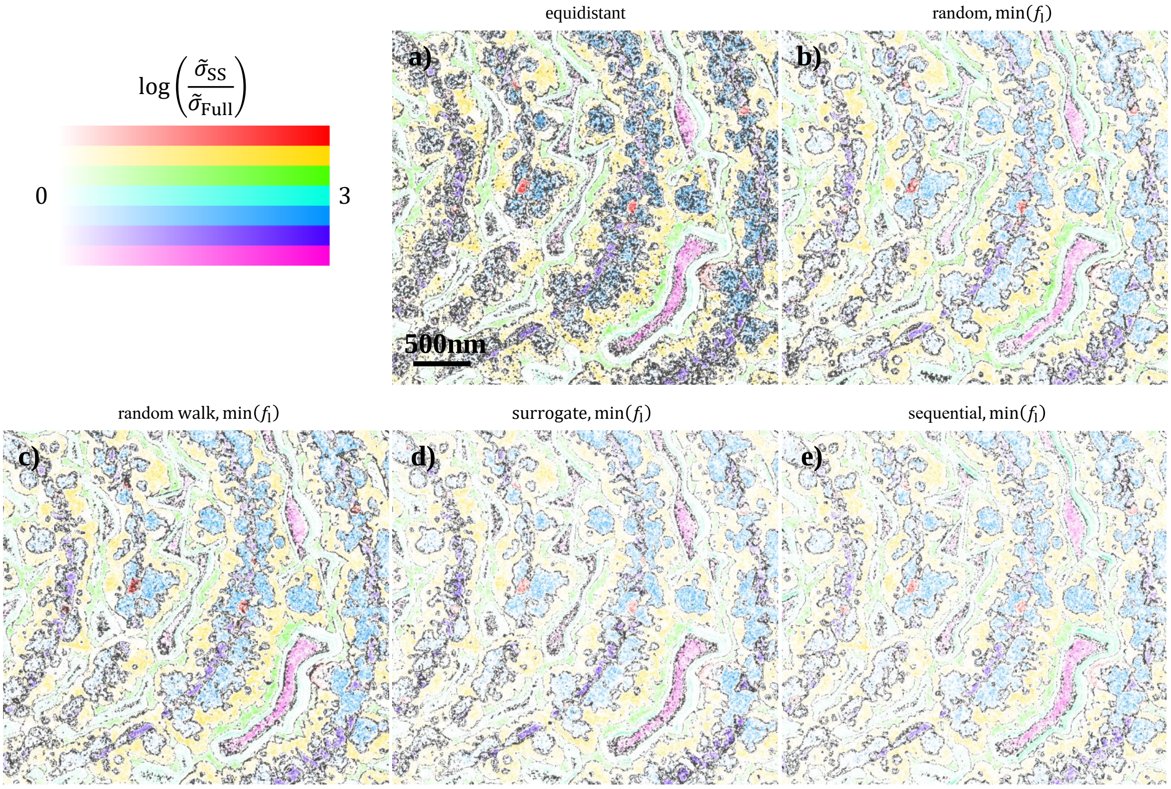

Figure S18: Loss of classification between sparse sampling and full spectral information, calculated as $l = \log\left(\frac{\tilde{\sigma}_{SS}}{\tilde{\sigma}_{Full}}\right)$, where the subscript indicates if the $\tilde{\sigma}$ is obtained from sparse sampling (SS) or the full spectral information method (Full), is encoded as saturation, given by $\min(0, \max(1 - l/3, 0))$. The hue encodes the classification index (colour code as Fig. 3). If the sparse sampling and full spectral classifications result into different classes, the hue is changed to dark grey. The results from different sampling methods are plotted in a) equidistant, b) random, c) random walk, d) surrogate optimisation and e) sequential selection.

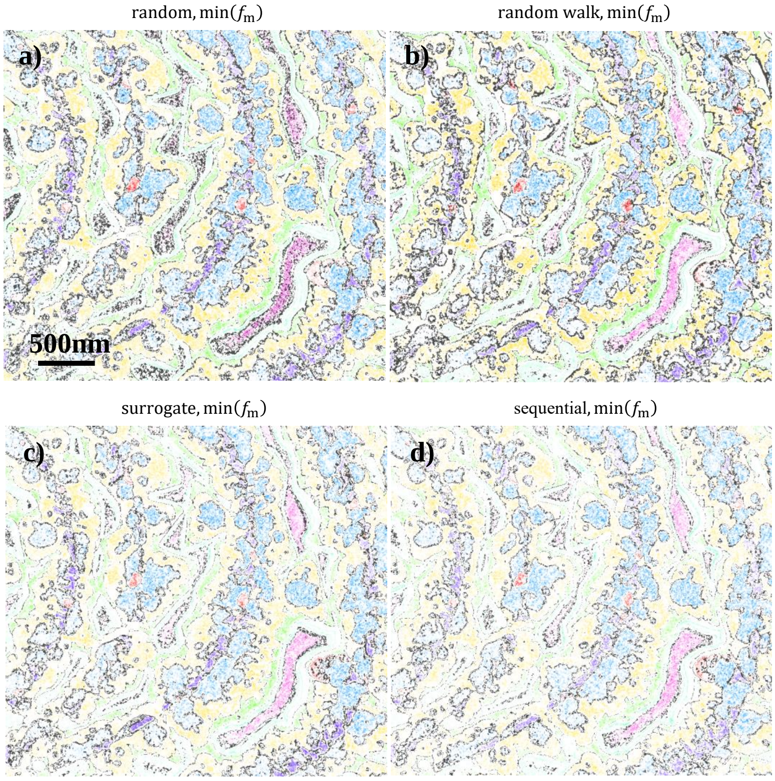

Figure S19: Same as Fig. S18 for the minimisation of f_m. The results from different sampling methods are plotted in a) random, b) random walk, c) surrogate optimisation and d) sequential selection.

S6 µ-LEED results

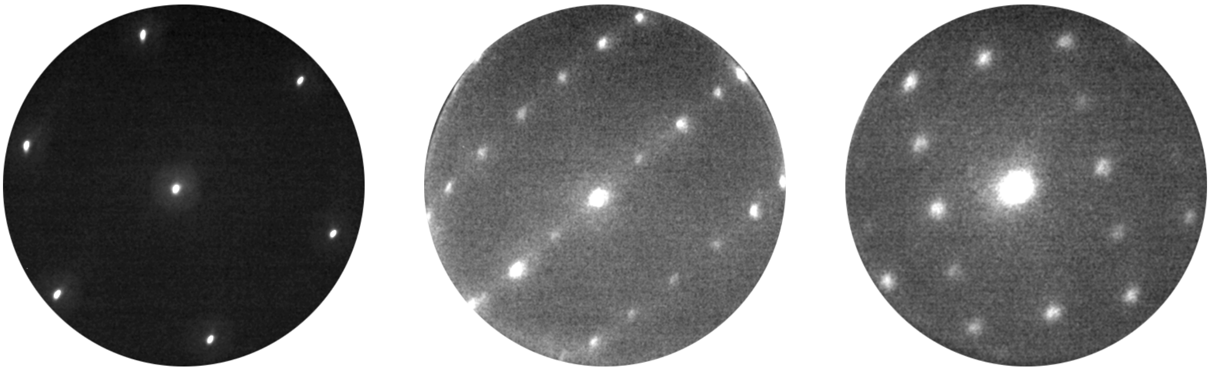

Figure S20: Low-energy electron diffraction (µ-LEED) patterns taken from specific regions on the RuO_2 sample presented in the main manuscript. A 250 nm diameter illumination aperture was used. a) (1×1) reconstructed single oxygen monolayer on Ru(0001) (recorded at 40 eV electron energy), b) $RuO_2(110)$/ Ru(0001) island (86 eV) and c) $RuO_2(101)$/ Ru(0001) island (55 eV).
